# Histone acetylation by HBO1 (KAT7) activates Wnt/β-catenin signaling to promote leukemogenesis in B-cell acute lymphoblastic leukemia

**DOI:** 10.1038/s41419-023-06019-0

**Published:** 2023-08-04

**Authors:** Hao Wang, Yingqi Qiu, Honghao Zhang, Ning Chang, Yuxing Hu, Jianyu Chen, Rong Hu, Peiyun Liao, Zhongwei Li, Yulu Yang, Qingyan Cen, Xiangyang Ding, Meifang Li, Xiaoling Xie, Yuhua Li

**Affiliations:** 1 Department of Hematology, Zhujiang Hospital, Southern Medical University, Guangzhou, Guangdong 510282 P. R. China; 2 Bioland Laboratory (Guangzhou Regenerative Medicine and Health Guangdong Laboratory), Guangzhou, Guangdong 510005 P. R. China

**Keywords:** Haematological cancer, Preclinical research, Acute lymphocytic leukaemia

## Abstract

B-cell acute lymphoblastic leukemia (B-ALL) is an aggressive hematological disorder with a dismal prognosis. The dysregulation of histone acetylation is of great significance in the pathogenesis and progression of B-ALL. Regarded as a fundamental acetyltransferase gene, the role of HBO1 (lysine acetyltransferase 7/KAT7) in B-ALL has not been investigated. Herein, we found that HBO1 expression was elevated in human B-ALL cells and associated with poor disease-free survival. Strikingly, HBO1 knockdown inhibited viability, proliferation, and G1-S cycle progression in B-ALL cells, while provoking apoptosis. In contrast, ectopic overexpression of HBO1 enhanced cell viability and proliferation but inhibited apoptotic activation. The results of in vivo experiments also certificated the inhibitory effect of HBO1 knockdown on tumor growth. Mechanistically, HBO1 acetylated histone H3K14, H4K8, and H4K12, followed by upregulating CTNNB1 expression, resulting in activation of the Wnt/β-catenin signaling pathway. Moreover, a novel small molecule inhibitor of HBO1, WM-3835, potently inhibited the progression of B-ALL. Our data identified HBO1 as an efficacious regulator of CTNNB1 with therapeutic potential in B-ALL.

## Introduction

B-cell acute lymphoblastic leukemia (B-ALL) is a hematologic malignancy with abnormal proliferation and accumulation of malignant B-lymphoid cells in the bone marrow, and its incidence accounts for 85% and 75% of ALL in children and adults, respectively [1, 2]. Currently, the 5-year overall survival rate of pediatric ALL patients is >90% [3, 4], but <45% of adults [5], and the prognosis is worse in patients older than 50 years, with a 5-year overall survival rate of less than 25% [3]. Chemotherapy and hematopoietic stem cell transplantation are currently the frontline treatments for B-ALL, but patients who relapse have a very poor prognosis [6]. Emerging novel therapeutics such as chimeric antigen receptor-T cells have achieved considerable efficacy in refractory/relapsed B-ALL (r/r B-ALL) but are still accompanied by a high recurrence rate [7–9]. As a result, exploring novel molecular pathogenesis and targeted therapy has become a focus of current B-ALL research [10–13].

Epigenetic mechanisms, such as histone acetylation and methylation, are thought to play an important role in the pathogenesis and progression of B-ALL [14–16], and histone modification via reversible acetylation is crucial for gene expression regulation [17]. Histone acetyltransferase (HAT) and histone deacetylase (HDAC) are two enzymes responsible for histone acetylation. Dysregulation of histone acetylation is closely associated with the progression of B-ALL [14, 18, 19].

HBO1 (also known as KAT7, MYST2) is a member of the MYST acetyltransferase family [20–22]. Lacking chromatin binding regions, it needs to form complexes with scaffold proteins (BRPF or JADE) to function in acetylating histones [23]. The HBO1-BRPF complex acetylates histone H3 tails (K14 and K23) [21, 23, 24], whereas the HBO1-JADE complex acetylates histone H4 tails (K5, K8, and K12) [25]. HBO1 is involved in various physiological processes such as DNA replication, gene transcription, protein ubiquitination, immune regulation, stem cell pluripotency, as well as embryonic development [22, 26].

Recent studies have shown that HBO1 facilitated the progression of cancers. Gao et al. demonstrated that HBO1 functioned as a novel oncogenic gene in osteosarcoma [27]. In hepatocellular carcinoma, overexpression of HBO1 could boost its progression in vitro and in vivo [28]. HBO1 acts as an oncogene to promote bladder cancer proliferation through activation of the Wnt/β-catenin signaling pathway, but the specific mechanism has not been fully investigated [29]. However, a study by Sauer et al. reported that HBO1 expression and histone H4 lysine acetylation were suppressed in acute myeloid leukemia (AML), and loss of HBO1 accelerated leukemic growth and colony formation [30]. Kueh and colleagues claimed that HBO1 was dispensable for DNA replication and cell proliferation in 293 T (embryonal kidney), MCF7 (breast adenocarcinoma), and HeLa (cervix carcinoma) cells [31]. So far, there have been no reports on the expression and function of HBO1 in B-ALL. This study shows that HBO1 is a potential therapeutic target for B-ALL.

## Materials and methods

### Chemicals and reagents

Cell Counting Kit-8 (CCK8) was obtained from Dojindo Co (Kumamoto, Japan). Puromycin was provided by Solarbio (Beijing, China). Antibodies for Tubulin (66031–1-Ig), HBO1 (13751–1-AP), Histone H4 (16047–1-AP), BCL2 (12789–1-AP), P21 (10355–1-AP), Cyclin D1 (60186–1-Ig), CDK4 (11026–1-AP), LaminB1 (66095–1-Ig), β-catenin (51067–2-AP), c-MYC (10828–1-AP) and MMP7 (10374–2-AP) were obtained from Proteintech (Wuhan, China). Antibodies for Histone H3 (#4499), acetyl-Histone H3 at Lys14 (H3K14ac, #7627), and cleaved-poly (ADP-ribose) polymerase (PARP) (#5625) were provided by Cell signaling Tech China (Shanghai, China). Antibodies for acetyl-Histone H3 at Lys23 (H3K23ac, BS70005) and acetyl-Histone H4 at Lys8 (H4K8ac, BS74016) were provided by Bioworld (Nanjing China). An acetyl-Histone H4 at Lys5 (H4K5ac, bs-10721-R) was provided by Bioss (Beijing, China). An acetyl-Histone H4 at Lys12 (H4K12ac, ab177793) was provided by Abcam (Shanghai, China). Caspase inhibitors, Z-DEVD-FMK, and Z-VAD-FMK were provided by Glpbio (Shanghai, China). Blasticidin and the HBO1 inhibitor WM-3835 were provided by MedChemExpress (Shanghai, China). Transfection reagent Lipofectamine 3000 was provided by Thermo-Fisher Invitrogen (Shanghai, China).

### Cell lines and culture conditions

The B-ALL cells (NALM-6, REH, and RS4;11), T-ALL cell (Jurkat), AML cells (KG1a, NB4, MV4–11, and THP-1), and lymphoma cells (Raji, Daudi, Jeko-1, and NAMALWA) were maintained in our laboratory (Hematological Laboratory of Zhujiang Hospital). Cell lines were identified by short tandem repeat (STR) profiling and confirmed to be mycoplasma-free immediately before use. Cells over passage 15 were not used. All cell lines were cultured in RPMI 1640 medium supplemented with 10% fetal bovine serum at 37 °C with 5% CO_2_.

### Clinical samples

Bone marrow mononuclear cells (BMMC) and peripheral blood mononuclear cells (PBMC) were obtained from bone marrow aspirates from B-ALL patients as well as peripheral blood (PB) from healthy individuals using Ficoll-Paque density gradient centrifugation. CD34^+^ PBMC were obtained from healthy human peripheral blood by Ficoll-Paque density gradient centrifugation and CD34 MicroBead kit (Miltenyi Biotec, Auburn, CA, USA) following the manufacturer’s instructions. Isolated cells were verified to be >95% pure by fluorescence-activated cell sorting (FACS). Before carrying out the experiments, informed consent was obtained from all patients and healthy donors according to the 1975 Declaration of Helsinki revised in 1983. All research involving human samples was approved by the Institutional Review Board of Zhujiang Hospital.

### qRT-PCR

Total RNA was extracted by TRIzol reagents and was reversely transcribed into cDNA using a PrimeScript RT reagent kit (Vazyme, Nanjing, China). qRT-PCR was performed by using an SYBR Green Dye detection system (BIO-RAD). β-actin was utilized as the internal control, and the 2^−ΔΔCt^ method was used to quantify the mRNA levels of target genes. All the mRNA primers were verified and provided by IGE (Guangzhou, China) and listed in Table S1.

### Western blot analysis

As described in our previous studies [32], prepared cell samples were homogenized in protein lysate buffer and centrifugated at 12,000 r for 10 min at 4 °C to remove the precipitate. The concentration of protein was determined by a BCA protein assay kit (KeyGEN BioTECH, Jiangsu, China). After the addition of the loading buffer, 30 μg of each protein sample was electrophoresed and then transferred to PVDF membranes (0.2 μm; Millipore, Bedford, MA). Membranes were blocked in skim milk for 1 h and then were immunoblotted with anti-human antibodies overnight at 4 °C. After three times washing with TBST, the membranes were incubated with horseradish peroxidase (HRP)-conjugated secondary antibodies at room temperature for 1 h, and the HRP signal was detected by FDbio-Dura ECL luminescent solution (Fdbio science, Zhejiang, China).

### HBO1 shRNA

Genechem (Shanghai, China) designed and validated two non-overlapping shRNA targeting human HBO1, including sh1 (target DNA sequence: CTGTCACCTGATTGGATATTT) and sh2 (target DNA sequence: GCTCAAATACTGGAAGGGAAA), as well as a scramble control shRNA (“shC”, target DNA sequence: TTCTCCGAACGTGTCACGT). The shRNAs were subcloned into the GV493 (hU6-MCS-CBh-gcGFP-IRES-puromycin) vector (Genechem), and the two constructs were transfected into HEK-293T cells separately with lentiviral packaging plasmid mixture (Genechem). B-ALL cells were seeded into 24-well plates (1 × 10^5^ cells per well) in a polybrene-containing complete medium. HBO1 shRNA or scramble control shRNA lentivirus was added to cultured B-ALL cells. The virus-containing medium was replaced with a fresh complete medium after 24 h. To select stable cells, puromycin (3.0 µg/mL) was added to the complete medium, and cells were cultured for 10 days. HBO1 knockdown in stable cells was assessed by qRT-PCR and western blot assays.

### HBO1 knockout

A single guide RNA (sgRNA) targeting human HBO1 (target DNA sequence: GATGAACGAGTCTGCCGAAG) or Control vector (“Control”, target DNA sequence: GTATTACTGATATTGGTGGG) was inserted into a lenti-CRISPR-GFP plasmid. B-ALL cells were seeded into 24-well plates containing complete medium (1 × 10^5^ cells/well). The constructs were transfected into B-ALL cells by Lipofectamine 3000. GFP-positive B-ALL cells were FACS sorted and then distributed into 96-well plates to form single-cell clones. Stable HBO1 knockout cells were then screened by qRT-PCR and western blot analysis.

### Ectopic HBO1 overexpression

The HBO1 cDNA sequence was synthesized and subcloned into the CV547 (Ubi-MCS-3FLG-SV40-Cherry-IRES-Blasticidin) gene expression lentiviral vector (Genechem). The construct and lentiviral packaging plasmid mixture were co-transfected into HEK-293T cells to generate lentivirus expressing HBO1. B-ALL cells were seeded into 24-well plates (1 × 10^5^ cells per well) in a polybrene-containing complete medium followed by adding lentivirus. The virus-containing medium was replaced with a fresh complete medium after 24 h. To select stable cells, blasticidin (3.0 µg/mL) was added to the complete medium, and cells were cultured for 10 days. Overexpression of HBO1 in stable cells was verified by qRT-PCR and western blot assays.

### Cell viability and death assays

For cell viability assays, cells were seeded into 96-well plates at 1 × 10^4^ cells per well and cultured. Then 10 μL CCK8 reagent was added to each well and incubated for 4 h. Then, the optical density (OD) value was recorded at 450 nm. For apoptosis detection experiments, genetically modified or treated B-ALL cells were seeded into six-well plates (2 × 10^5^ cells per well) and cultured. Cells were then collected to remove the complete medium and washed twice with PBS before being stained with Annexin V and propidium iodide (PI) (both at 10 μg/mL; KeyGEN BioTECH). Cells were then subjected to FACS for 1 h (Beckman Coulter, Brea, CA). Annexin V-positive cells were gated and considered apoptotic cells. For trypan blue staining, the medium was removed after collecting the cells and then resuspended with PBS. Then draw 90 µL cell suspension and add 10 μL trypan blue staining solution, mix well, and stain for 1 min, then observe the proportion of blue cells under an inverted light microscope.

### EdU (5-ethynyl-20-deoxyuridine) staining

Briefly, B-ALL cells were seeded into six-well plates at 3 × 10^5^ cells per well and cultured. The cell proliferation was quantified by an EdU Apollo-567 kit (RiboBio, Guangzhou, China) according to the protocol. Following adding EdU and Hoechst 33342 fluorescent dyes, B-ALL cells were visualized under a fluorescent microscope (Nikon, Shanghai, China). Three random views with a total of over 300 cells were used to calculate the cell proliferation ratio.

### Colony formation assays

Cells were seeded in a 12-well plate at a density of 1000 cells per well. Methylcellulose (Sigma-Aldrich, USA) was added at a final concentration of 0.9% and mixed with the cell suspension. The number of colonies was calculated under the microscope after 10 days of incubation.

### Cell cycle FACS

1 × 10^6^ B-ALL cells were collected and centrifuged to remove the medium and washed twice with PBS. After resuspension with 75% ethanol and incubation at 4 °C overnight, ethanol was removed by centrifugation and washed twice with PBS. Finally, cells were stained using 0.5 mL PI/RNase Staining Buffer (BD biosciences, USA) and incubated at room temperature for 15 min followed by FACS analysis within 1 h.

### ssDNA ELISA

Gene-modified or treated B-ALL cells were seeded into six-well plates (1 × 10^6^ cells per well) and cultured. Cells collected by centrifugation were washed three times with cold PBS. Then cells were resuspended in 200 μL PBS and disrupted by repeated freezing and thawing. The extract was centrifuged at 1500 × *g* for 10 min, and the supernatant was taken for detection according to the protocol of an ssDNA ELISA kit (RUIXIN BIOTECH, Fujian, China). The absorbance was detected at 450 nm.

### Caspase-3 activity

Gene-modified or treated B-ALL cells were seeded into six-well plates (3 × 10^5^ cells per well) and cultured. Then, cells were collected and lysed with cell lysis buffer. According to the caspase-3 activity assay kit protocol (Beyotime, Shanghai, China), caspase-3 could catalyze the substrate acetyl-Asp-Glu-Val-Asp p-nitroanilide (Ac-DEVD-pNA) to produce yellow p-nitroaniline (pNA), so the activity of caspase-3 could be detected by measuring the absorbance. pNA showed strong absorption near 405 nm.

### TOP/FOP flash activity assays

The wild-type (TOP) and mutant (FOP) LEF/TCF reporters were cloned into the pGL6-TA luciferase construct (Beyotime). B-ALL cells were seeded in triplicate in 96-well plates (2 × 10^4^ cells per well). 100 ng of TOP or FOP flash was transfected into cells along with 1 ng of pRL-TK Renilla plasmid (Beyotime) using the lipofectamine 3000 reagent. After 48 h, a Dual-luciferase Reporter Gene Assay Kit (Beyotime) was used to detect the luciferase and Renilla signals according to the protocol provided by the manufacturer.

### Plasmids and cell transfection

The pCDNA3.1(+)-Homo-β-catenin-NLS, pCDNA3.1(+)-MYC-Homo-HBO1-NLS and pCDNA3.1(+)-MYC-Homo-HBO1-G485A-NLS plasmids were provided by IGE. B-ALL cells were seeded into 6-well plates containing complete medium (1 × 10^6^ cells/well). These plasmids were transfected into B-ALL cells with Lipofectamine 3000 and then the expression was verified through western blot assays.

### RNA-seq

Total RNA from NALM-6/shC and NALM-6/shHBO1 was extracted with TRIzol reagent, and sequencing was performed in the Illumina NovaSeq platform. To identify differentially expressed genes by RefSeq ID, the DESeq2-EBSeq package was used. The differential gene expression analysis was performed using BMKCloud. Sequencing data have been uploaded to the GEO database: GSE233678.

### Xenograft assays

The NOD.CB17-Prkdcscid/l2rgtm1/Bcgen (B-NGD) mice (4–6 weeks, female) were purchased from BIOSYTOGEN (Jiangsu, China) and maintained under specific pathogen-free (SPF) conditions. For subcutaneous xenograft models, 5 × 10^6^ shHBO1 NALM-6 or control cells were subcutaneously (s.c.) injected into the right flank of mice (*n* = 5 per group). Tumor volumes were recorded every five days according to the formula: (length × width^2^)/2. On day 25, tumor tissues were removed from all mice following euthanasia to perform immunohistochemistry and western blot experiments. For the HBO1 knockdown tail vein xenograft model, 2 × 10^5^ GFP-tagged NALM-6-shHBO1 or NALM-6-shC cells were injected into B-NDG mice via the tail vein, and the mice were sacrificed 20 days later to remove the spleens and bone marrows for FACS analysis of tumor burden (*n* = 5 per group). In addition, survival times of tumor-bearing mouse models constructed by tail vein injection of NAM-6-shC or NALM-6-shHBO1 were recorded (*n* = 8 per group). To observe the anti-tumor effect of WM-3835 in vivo, 2 × 10^5^ GFP-labeled wild-type NALM-6 cells were injected into B-NDG mice via the tail vein (day 1), and 7 days later, WM-3835 (at 10 mg/kg body weight, daily for 2 weeks) or vehicle control was injected intraperitoneally, and on day 20, the mice were sacrificed and the spleens and bone marrows were removed for FACS analysis to observe the tumor burden (*n* = 5 per group). Survival time of tumor-bearing mice was documented following treatment with WM-3835 or vehicle control (*n* = 8 per group). All animal studies were approved by the Ethical Committee for Animal Research of Zhujiang Hospital of Southern Medical University.

### Chromatin CHIP-qPCR assays

B-ALL cells were processed to ChIP assays using SimpleChIP Kits (#9003; Cell Signaling, Danvers, MA, USA) according to the manufacturer’s instructions. The HBO1, H3K14ac, H4K8ac, or H4K12ac antibodies were added to form the antibody-target protein-DNA complex. DNA samples obtained after immunoprecipitation were undergoing qPCR analysis. CTNNB1 gene promotor-specific primers are listed in Table S1. Anti-IgG antibody was used as a negative control.

### Statistical analysis

All the experimental assays in this study were repeated at least three times. Data were analyzed by Graphpad Prism 9 software and results were presented as mean ± standard deviation (SD). Data were analyzed by *t* test between two groups and ANOVA and Dunnett’s test between multiple groups. Survival curves were analyzed by the Kaplan–Meier method and compared by the log-rank test. Statistical significance was determined as *P < 0.05, **P < 0.01, ***P < 0.001, ****P < 0.0001, or no significance (ns).

## Results

### HBO1 is overexpressed in B-ALL

**Fig. 1:**
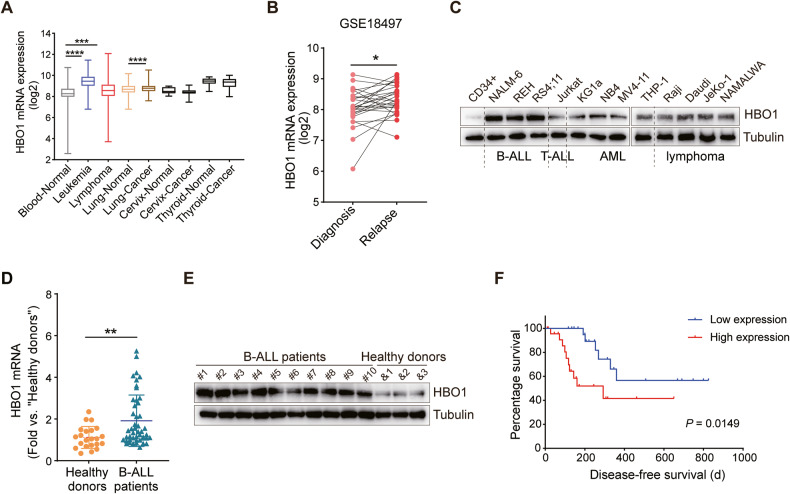
HBO1 upregulation in B-ALL. **A** GENT2 database (link: http://gent2.appex.kr/gent2/) showed the expression of HBO1 mRNA in different cancers. **B** Expression levels of HBO1 mRNA in matched diagnosis-relapse B-ALL patients from the GEO database (link: https://www.ncbi.nlm.nih.gov/geo/GSE18497). **C** HBO1 protein expression in PB CD34^+^ cells from healthy donors and a panel of malignant hematological cells. **D**, **E** HBO1 mRNA (**D**) and protein expression (**E**) in BMMC of B-ALL patients and PBMC of healthy donors in our clinical samples. **F** Kaplan–Meier survival analyses of HBO1-low expression and HBO1-high expression B-ALL patients in our clinical sample data. Error bars indicate mean ± standard deviation (SD). Significance was tested by one-way ANOVA and two-tailed unpaired student’s *t* tests (**A**, **D**), two-tailed paired student’s *t* tests (**B**), and log-rank test (**F**). *P < 0.05, **P < 0.01, ***P < 0.001, ****P < 0.0001.

Firstly, we searched the GENT2 database to examine the expression level of HBO1 mRNA in leukemia, lymphoma, and a variety of other tumors as compared with corresponding normal tissues. As demonstrated, the expression of HBO1 mRNA was significantly increased in patients with leukemia, lymphoma, or lung cancer, but not in patients with cervix or thyroid cancer (Fig. 1A). Results from the GEO database showed that HBO1 mRNA expression in B-ALL patients during recurrence is higher than that during diagnosis. (Fig. 1B).

To validate the bioinformatics results, we examined the expression of HBO1 protein in CD34^+^ cells from healthy human peripheral blood and various ALL, AML, and lymphoma cell lines. As expected, the expression of HBO1 protein in malignant hematological cell lines was significantly higher than that in CD34^+^ cells. Interestingly, the expression of HBO1 protein in B-ALL cell lines (NALM-6, REH, and RS4;11) was significantly higher than that in T-ALL cell (Jurkat), AML cell (KG1a, NB4, MV4-11, and THP-1), and lymphoma cell lines (Raji, Daudi, Jeko-1, and NAMALWA) (Fig. 1C).

To examine the clinical relevance of HBO1, PBMC from healthy donors and BMMC from B-ALL patients were collected. Table S2 described the clinical characteristics of B-ALL patients. We performed qRT-PCR to detect the expression level of HBO1 mRNA. As shown in Fig. 1D, HBO1 mRNA expression was higher in B-ALL patients than that in healthy donors. We also examined whether there was a relationship between HBO1 expression levels and mutations in BCR-ABL, IKZF1, and WT1 genes, and the results indicated that HBO1 expression levels in patients carrying these mutations were not higher than those in patients without mutations (Fig. S1). Utilizing western blot analysis, we confirmed the dramatic HBO1 protein upregulation in B-ALL samples (Fig. 1E). Furthermore, HBO1 overexpression is associated with poor disease-free survival in B-ALL patients (Fig. 1F). These results demonstrated that HBO1 was upregulated in B-ALL and associated with poor survival.

### HBO1 shRNA inhibits B-ALL cell viability, proliferation, and cell cycle progression

**Fig. 2:**
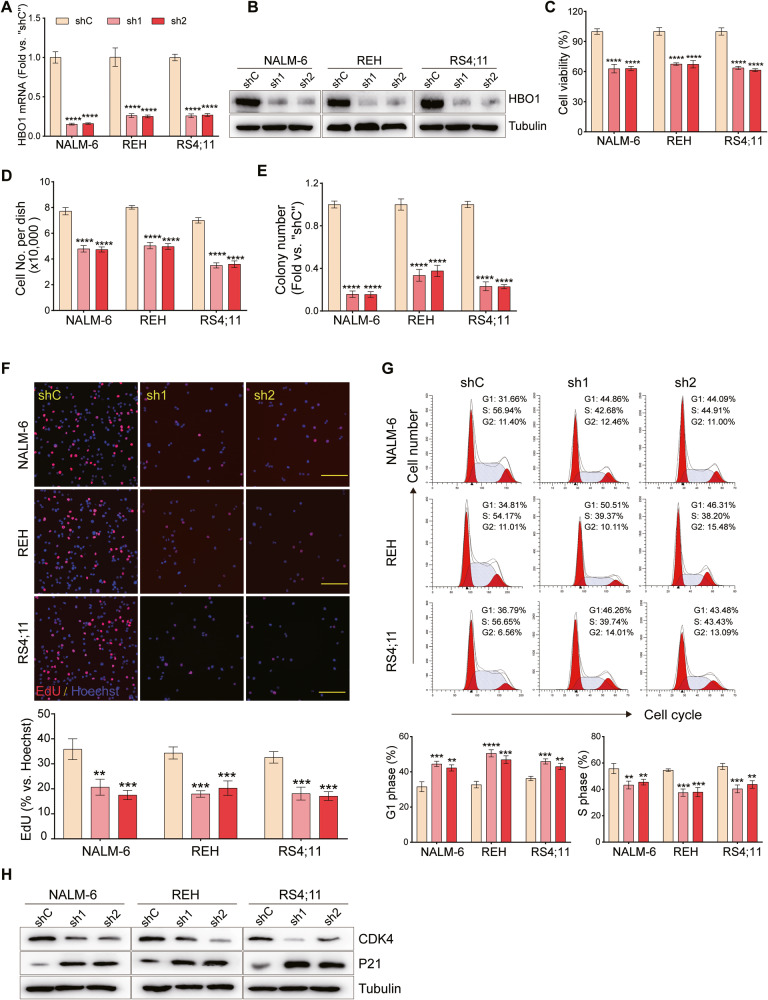
HBO1 shRNA inhibits B-ALL cell viability, proliferation, and cell cycle progression. **A**, **B** B-ALL cells, stably expressing control shRNA (“shC”), or HBO1 shRNA (“sh1/2”, with no-overlapping sequences) were cultured in the complete medium and detected the expression of HBO1 mRNA (**A**) and protein (**B**). **C** CCK8 assays were used to analyze the cell viability of B-ALL cells with or without HBO1 knockdown cultured in the medium for 96 h. **D** Cell counting was performed to observe the proliferation of B-ALL cells treated as **C**. **E** Colony formation of B-ALL cells cultured for 10 days with or without HBO1 knockdown. **F** Nuclear EdU incorporation (upper) and statistics (lower) of B-ALL cells cultured for 48 h with or without HBO1 knockdown. **G** FACS analysis of cell cycle progression (upper) and statistics (lower) in B-ALL treated as **F**. **H** Western blot assays were performed to detect the expression of cell cycle-related proteins after the knockdown of HBO1. Scale bar = 100 μm (**F**). Error bars indicate mean ± standard deviation (SD). Significance was tested by one-way ANOVA (**A**, **C**–**G**). **P < 0.01, ***P < 0.001, ****P < 0.0001. *n* = 3 per group (**A**, **C**–**G**).

To study the potential function of HBO1 in B-ALL cells, two lentiviral shRNAs (with GFP and puromycin selection gene) targeting non-overlapping sequences of human HBO1 (sh1/2) were individually transfected into NALM-6, REH, and RS4;11 cells. Following puromycin selection, stable cells were established. qRT-PCR analysis demonstrated that HBO1 mRNA decreased by >75% in stable B-ALL cells with HBO1 shRNA (Fig. 2A). The HBO1 protein was robustly downregulated as well (Fig. 2B). As compared to the cells transfected with scramble control shRNA (“shC”), viability was significantly reduced in HBO1 knockdown cells (Fig. 2C). Results in Fig. 2D demonstrated that the proliferation of HBO1 knockdown cells was largely hindered. Moreover, HBO1 knockdown significantly inhibited colony formation (Fig. 2E) and nuclear EdU incorporation (Fig. 2F), also indicating proliferation inhibition.

Previous studies suggested that HBO1 was associated with some key events of cell cycle progression, especially in mitosis through interaction with CDK1 and PLK1 [33]. The FACS assays showed that HBO1 knockdown increased the number of cells in the G1 phase and decreased the number of cells in the S phase (Fig. 2G). CDK4 is a key mediator of the cellular transition to the S phase and is important for the initiation, growth, and survival of several cancer types, including B-ALL [34, 35]. P21 is also a cell cycle regulator involved in modulating cell cycle arrest [36]. Through western blot experiments, we found that the expression of CDK4 protein decreased after HBO1 knockdown, accompanied by an increase in P21 protein expression (Fig. 2H). Thus, HBO1 knockdown induced G1-S cell cycle arrest in B-ALL cells. Overall, these results suggested that HBO1 knockdown inhibited the viability, proliferation, and cell cycle progression of B-ALL cells.

### HBO1 knockdown provokes apoptosis of B-ALL cells

**Fig. 3:**
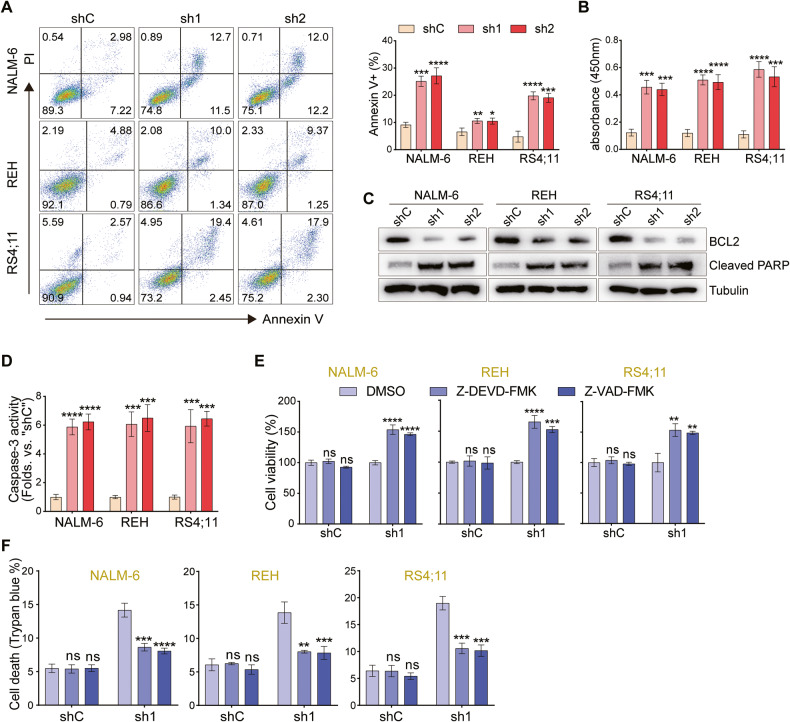
HBO1 knockdown provokes apoptosis of B-ALL cells. **A** FACS analysis (left) and statistics (right) of the apoptosis of B-ALL cells cultured for 48 h with or without HBO1 knockdown. **B** The contents of ssDNA in B-ALL cells treated as **A**. **C** The expression of apoptosis-related proteins in B-ALL cells with or without HBO1 knockdown. **D** The statistical plot of caspase-3 activity in B-ALL cells treated as **A**. **E**, **F** B-ALL cells with or without HBO1 knockdown were treated with Z-DEVD-FMK (30 μM), Z-VAD-FMK (30 μM), or vehicle (0.1% of DMSO) and cultured for 96 h, followed by the cell viability (**E**) and death (**F**) examination by CCK8 and trypan blue staining. Error bars indicate mean ± standard deviation (SD). Significance was tested by one-way ANOVA (**A**, **B**, **D**–**F**), *P < 0.05, **P < 0.01, ***P < 0.001, ****P < 0.0001, ns no significance. *n* = 3 per group (**A**, **B**, **D**–**F**).

RNA-seq analysis in one study identified more than 250 differentially regulated genes in HBO1-depleted cells, some of which are anti-apoptosis genes [37]. In addition, recent studies have also manifested apoptotic activation of AML cells in the absence of HBO1 [38]. Herein, the potential impact of HBO1 deficiency in B-ALL cell apoptosis was investigated. A substantially increased number of Annexin V-positive cells was observed after HBO1 shRNA transfection (Fig. 3A). As shown in Fig. 3B, in HBO1 knockdown cells, the contents of ssDNA significantly increased, indicating increased DNA breakage.

Besides, western blot assays showed that HBO1 shRNA downregulated the expression of the anti-apoptotic protein BCL2 and promoted the cleavage of the PARP protein (Fig. 3C). Caspase-3 activity assays unveiled that HBO1 knockdown led to the increase of caspase-3 activity, further confirming the activation of apoptosis (Fig. 3D). Viability reduction and cell death induced by shHBO1 were significantly attenuated after the use of two caspase inhibitors, the caspase-3 inhibitor Z-DEVD-FMK and the pan-caspase inhibitor Z-VAD- FMK (Fig. 3E, F). Taken together, HBO1 knockdown provoked apoptosis in B-ALL cells.

### HBO1 knockout by CRISPR/Cas9 induces significant anti-B-ALL cell activity

To exclude possible off-target effects of HBO1 shRNA application, we generated a CRISPR/Cas9 vector containing HBO1 sgRNA and transduced it into NALM-6 and REH cells, and then, FACS-mediated GFP sorting was used to establish stable monoclonal HBO1 knockout cells. HBO1 protein expression was almost completely abolished compared to control cells transfected with CRISPR/Cas9 control vector (“control”) (Fig. S2A). Functional studies demonstrated that CRISPR/Cas9-induced HBO1 knockout largely reduced cell viability (Fig. S2B) and inhibited proliferation (Fig. S2C, E). Furthermore, HBO1 knockout markedly increased apoptosis in NALM-6 and REH cells, as shown by increased caspase-3 activity (Fig. S2D) as well as the proportion of Annexin V+ cells (Fig. S2F). Therefore, CRISPR/Cas9-induced HBO1 knockout produced significant anti-B-ALL cell activity.

### Ectopic HBO1 overexpression promotes B-ALL cell progression in vitro

**Fig. 4:**
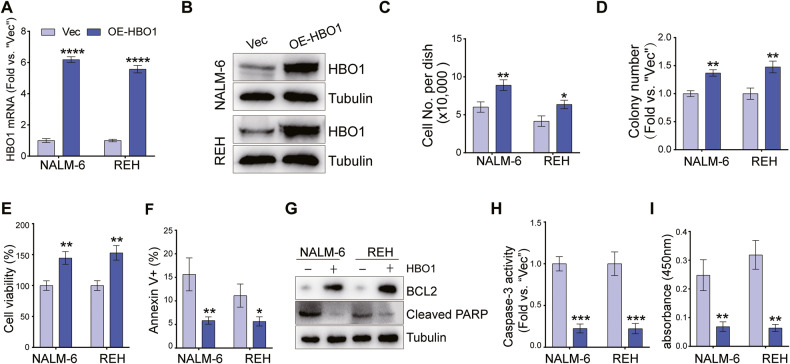
Ectopic HBO1 overexpression promotes B-ALL progression in vitro. **A** The expression level of HBO1 mRNA in NALM-6 and REH cells overexpressing HBO1. **B** The expression of HBO1 protein in B-ALL cells after overexpression of HBO1. **C** Cell number of NALM-6 and REH cells cultured for 96 h with or without HBO1 overexpression. **D** Colony formation of NALM-6 and REH cells cultured for 10 days with or without HBO1 overexpression. **E** The viability of NALM-6 and REH cells treated as **C**. **F** The proportion of apoptosis cells was analyzed by FACS in NALM-6 and REH cells cultured for 48 h with or without HBO1 overexpression. **G** Apoptosis-related proteins were detected by western blot assays in NALM-6 and REH cells treated as **F**. **H** Caspase-3 activity of NALM-6 and REH cells treated as **F**. **I** ssDNA content of NALM-6 and REH cells treated as **F**. Error bars indicate mean ± standard deviation (SD). Significance was tested by two-tailed unpaired student’s *t* tests (**A**, **C**–**F**, **H**–**I**), *P < 0.05, **P < 0.01, ***P < 0.001, ****P < 0.0001. *n* = 3 per group (**A**, **C**–**F**, **H**–**I**).

Since HBO1 silencing inhibited B-ALL cell growth and induced apoptosis significantly, we proposed that ectopic overexpression of HBO1 would have the opposite effect. To verify this hypothesis, we transfected an HBO1-expressing construct (OE-HBO1) into NALM-6 and REH cells, and stable OE-HBO1 cells were established through blasticidin selection. Compared with cells transfected with the control vector (“Vec”), the mRNA level of HBO1 increased (over 5 folds) in OE-HBO1 cells (Fig. 4A), accompanied by the upregulation of HBO1 protein (Fig. 4B). OE-HBO1 promoted the proliferation and colony formation of NALM-6 and REH cells (Fig. 4C, D). CCK8 assays confirmed that ectopic overexpression of HBO1 enhanced the viability of B-ALL cells (Fig. 4E). Ectopic overexpression of HBO1 reduced the number of Annexin V-positive cells (Fig. 4F), along with an increased expression of anti-apoptotic protein BCL2 and a decrease in cleaved PARP (Fig. 4G). Additionally, OE-HBO1 also reduced caspase-3 activity (Fig. 4H) and the break of DNA (Fig. 4I). These results implied that ectopic overexpression of HBO1 promoted proliferation but inhibited the apoptosis of B-ALL cells, further certificating the oncogenic role of HBO1 in B-ALL.

### HBO1 upregulates CTNNB1 expression in B-ALL to activate Wnt/β-catenin signaling

**Fig. 5:**
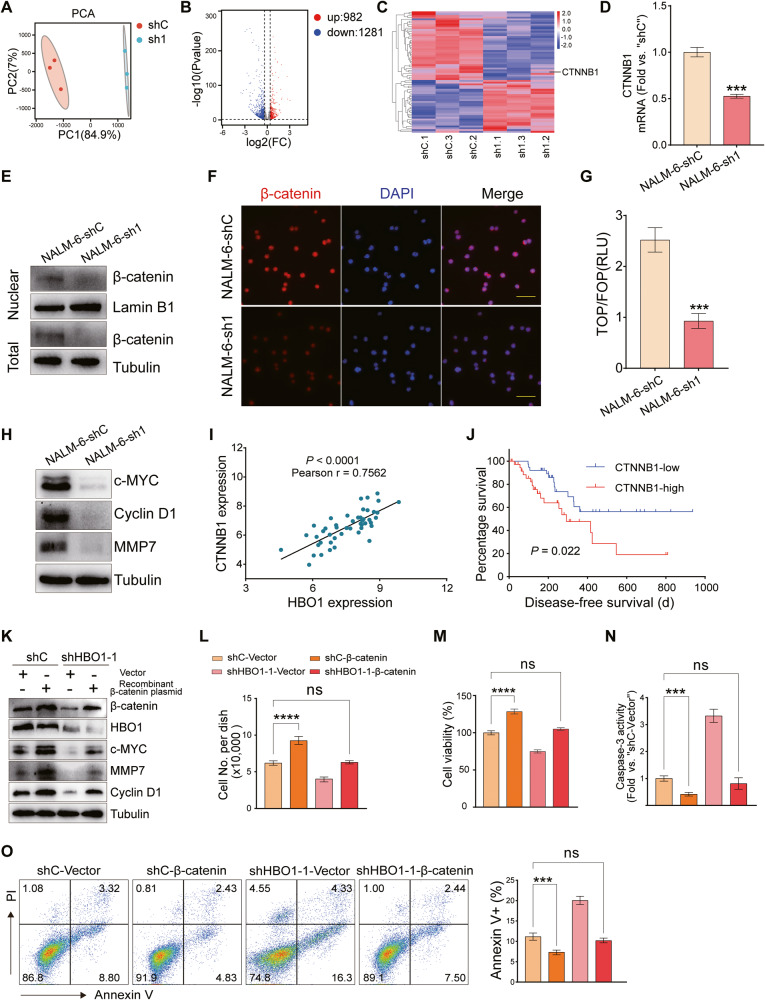
HBO1 upregulates CTNNB1 expression in B-ALL to activate Wnt/β-catenin signaling. **A** Principal component analysis of RNA sequencing data in NALM-6 cells with or without HBO1 knockdown. **B** Volcano plot showing differential genes of NALM-6 cells treated as **A**. **C** Heatmap of the gene expression pattern of Wnt/β-catenin signaling pathway in NALM-6 cells treated as **A**. **D** The expression level of CTNNB1 mRNA in NALM-6 cells with or without HBO1 knockdown. **E**, **F** Western blot (**E**) and immunofluorescence assays (**F**) were used to detect the expression of HBO1 protein in NALM-6 cells treated as **D**. **G** TOP/FOP flash assays were used to detect the activity of Wnt/β-catenin signaling in NALM-6 cells treated as **D**. **H** The expression of c-MYC, Cyclin D1, and MMP7 proteins in NALM-6 cells following HBO1 knockdown. **I** The expression relationship between HBO1 and CTNNB1 mRNA in clinical specimens. **J** Kaplan–Meier survival analyses of B-ALL patients with CTNNB1-low expression or CTNNB1-high expression in our sample data. **K** NALM-6-shC/shHBO1–1 cells were transfected with recombinant β-catenin plasmid or control vector plasmid (“vector”), followed by detecting the listed proteins. **L**, **M** Cell number (**L**) and viability (**M**) of NALM-6-shC/shHBO1–1 cells transfected with recombinant β-catenin plasmid or control vector and cultured for 96 h. **N** NALM-6-shC/shHBO1–1 cells were treated as **L**, then caspase-3 activity was determined. **O** FACS analysis (left) and statistics (right) of NALM-6-shC/shHBO1–1 cells treated as **L**. Scale bar = 100 μm (**F**). Error bars indicate mean ± standard deviation (SD). Significance was tested by two-tailed unpaired student’s *t* tests (**D**, **G**), nonlinear regression analysis (**I**), log-rank test (**J**), and one-way ANOVA (**L**–**O**), ***P < 0.001, ****P < 0.0001, ns significance. *n* = 3 per group (**D**, **G**, **L**–**O**).

To further explore the underlying mechanism by which HBO1 promotes B-ALL progression, we generated and performed comparative transcriptomic and functional analyses of shC *vs*. shHBO1 NALM-6 cells. Principal component analysis (PCA) showed a separation between the two groups (Fig. 5A). Knocking down HBO1 led to the upregulation of 982 genes and the downregulation of 1281 genes (*P* <0.05, Fig. 5B). HBO1 is a histone acetylation-modifying enzyme, and epigenetic modifications such as histone acetylation are closely related to the Wnt/β-catenin signaling pathway [39, 40]. In addition, HBO1 has been found to promote the development of bladder cancer and glioma by activating the Wnt/β-catenin signaling pathway [29, 41]. Therefore, we would like to further investigate the relationship between HBO1 and the Wnt/β-catenin signaling pathway in B-ALL. Among these transcripts, we found altered expression of 69 Wnt/β-catenin signaling pathway-related genes following HBO1 knockdown, including a gene encoding β-catenin protein (a key molecule of the Wnt pathway), CTNNB1 (Fig. 5C). To further confirm the results of RNA sequencing, qRT-PCR assays were conducted and manifested that the CTNNB1 mRNA expression level was decreased in NALM-6 and REH cells with HBO1 knockdown (Fig. 5D and S3A). Subsequently, western blot and immunofluorescence analyses were utilized to detect the expression of the β-catenin protein. Consistent with the mRNA results, a significant decrease of the β-catenin protein expression was found in NALM-6 and REH cells with HBO1 knockdown (Fig. 5E, F and S3B, C). The TOP/FOP Flash luciferase reporter assays were conducted to detect the Wnt/β-catenin signaling activity. Compared with control cells, shHBO1 significantly inhibited the TOP-Flash reporter activity in NALM-6 and REH cells (Fig. 5G and S3D). In addition, the expression of c-MYC, Cyclin D1, and MMP7 proteins, three downstream effectors of canonical Wnt/β-catenin signaling, were remarkably decreased after HBO1 knockdown (Fig. 5H and S3E). We also detected the correlation between the expression of HBO1 and CTNNB1 in clinical B-ALL specimens by qRT-PCR, and the results indicated that the mRNA expression level of CTNNB1 was positively correlated with that of HBO1 (Fig. 5I, *r* = 0.7562, *P* < 0.0001). Meanwhile, B-ALL patients with high CTNNB1 expression showed shorter disease-free survival (Fig. 5J). These results supported that HBO1 acted as a positive regulator of CTNNB1 in B-ALL.

To further validate that HBO1 regulates CTNNB1 expression in B-ALL cells, we overexpressed the recombinant β-catenin in NALM-6 cells previously transfected with shHBO1–1 or shC, which were examined via western blot. Compared with the control vector plasmid (“vector”), overexpression of β-catenin in NALM-6/shC cells increased the expression levels of c-MYC, MMP7, and Cyclin D1 proteins, except HBO1 protein (Fig. 5K). Simultaneously, NALM-6/shC cells transfected with β-catenin plasmid exhibited a higher proliferation rate, stronger viability, and lower apoptosis rate than cells transfected with an empty vector (Fig. 5L–O). Furthermore, β-catenin overexpression in NALM-6/shHBO1–1 cell markedly restored the expression of c-MYC, MMP7, and Cyclin D1 proteins, except HBO1 protein (Fig. 5K). As expected, overexpression of β-catenin in NALM-6/shHBO1–1 cells restored the decreased cell proliferation and viability and the activation of apoptosis caused by HBO1 knockdown (Fig. 5L–O). These data suggested that HBO1 upregulated CTNNB1 expression to activate the Wnt/β-catenin signaling pathway, resulting in B-ALL progression.

### HBO1 acetyltransferase activity is required for activating Wnt/β-catenin signaling

**Fig. 6:**
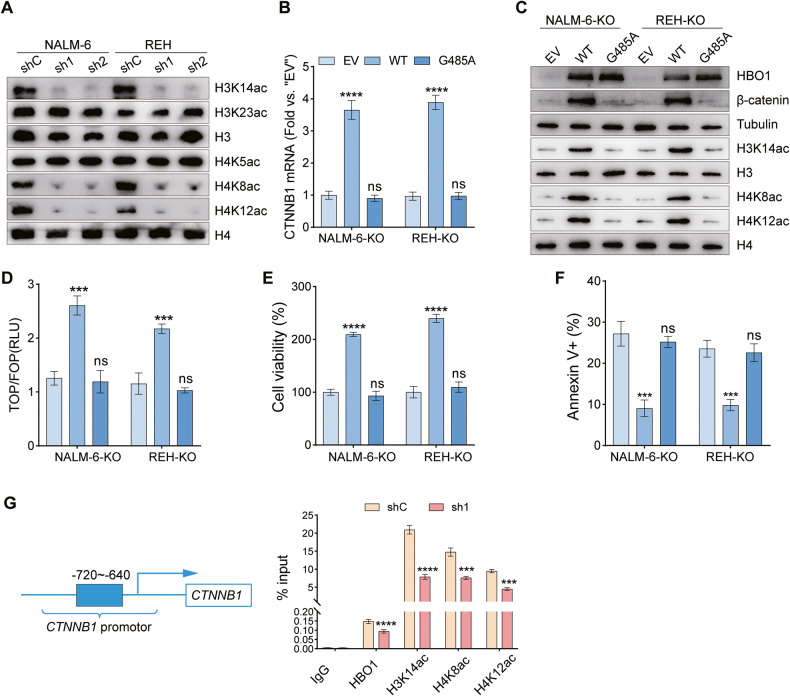
HBO1 acetyltransferase activity is required for activating Wnt/β-catenin signaling. **A** The expression of listed proteins in NALM-6 and REH cells transfected with HBO1-shC/shHBO1 lentivirus. **B**–**D** NALM-6 and REH cells with HBO1 knockout were transfected with wild-type (WT) HBO1 plasmid, G485A mutant or empty vector (EV) and cultured for 72 h, then CTNNB1 mRNA (**B**), listed proteins (**C**), and the activity of Wnt signaling (**D**) were tested. **E** Cell viability of NALM-6 and REH cells with HBO1 knockout and treated as **B**. **F** The proportion of apoptosis in NALM-6 and REH cells with HBO1 knockout and treated as **B**. **G** CHIP-qPCR assays were performed for NALM-6 cells treated with either control shRNA or HBO1 shRNA. Chromatin DNA was pulled down using respective antibodies of IgG, HBO1, acetylated H3K14, H4K8, and H4K12. Results showed that HBO1 knockdown ameliorated the binding of HBO1 and acetylation of H3K14 and H4K8/K12 on the promotor region of the CTNNB1 gene (−702~−640). Error bars indicate mean ± standard deviation (SD). Significance was tested by one-way ANOVA (**B**, **D**–**F**) and two-tailed unpaired student’s *t* tests (**G**), ***P < 0.001, ****P < 0.0001, ns significance. *n* = 3 per group (**B**, **D**–**G**).

HBO1 is known to form two different HAT complexes that acetylate either H3 or H4 [23]. To further determine potential mechanisms by which HBO1 regulates Wnt/β-catenin signaling, we detected the acetylation status of H3 and H4 tails in HBO1 knockdown NALM-6 and REH cells. Among the potential HBO1 acetylation sites, H3K14ac, H4K8ac, and H4K12ac were significantly decreased upon HBO1 knockdown, whereas other acetylation sites were not affected (Fig. 6A).

To examine whether HBO1 acetyltransferase activity is required for activating Wnt/β-catenin signaling, we generated an HBO1 acetyltransferase activity deficient mutant, HBO1-G485A [42]. Re-expression of HBO1 wild-type (HBO1-WT) in HBO1 knockout B-ALL cells promoted the expression of β-catenin at mRNA and protein levels, the acetylation of H3K14, H4K8, and H4K12 (Fig. 6B, C), as well as the activation of Wnt/β-catenin signaling pathway (Fig. 6D) compared with the empty vector (EV) group. Functional experiments showed that compared with the EV control, overexpression of the HBO1-WT significantly enhanced the viability of HBO1 knockout cells and reduced cell apoptosis (Fig. 6E, F). However, overexpression of HBO1-G485A mutant exerted no effects on β-catenin expression, H3K14, H4K8, and H4K12 acetylation, activation of Wnt/β-catenin signaling pathway, cell viability, and apoptosis in HBO1 knockout cells (Fig. 6B–F). The enrichment of acetylated histones in promoter regions is essential for regulating gene transcription [43, 44]. CHIP-qPCR assay using HBO1 antibody could determine the binding of HBO1 on the promotor region of CTNNB1. We found that HBO1 bound to the promoter region of CTNNB1, and HBO1 binding was reduced in HBO1 knockdown NALM-6 cells (Fig. 6G). As shown in Fig. 6A, the acetylation of H3K14, H4K8, and H4K12 is dependent on the expression of HBO1 in the B-ALL cells. We further carried out the CHIP-qPCR assays using H3K14ac, H4K8ac, and H4K12ac antibodies. The results showed that H3K14ac, H4K8ac, and H4K12ac were enriched in the promoter region of CTNNB1. Like HBO1, the binding of H3K14ac, H4K8ac, and H4K12ac was significantly decreased in HBO1 knockdown NALM-6 cells (Fig. 6G). These results suggested that HBO1 promoted CTNNB1 transcription by acetylating H3K14, H4K8, and H4K12, which was important for activating Wnt/β-catenin signaling.

### HBO1 knockdown suppresses B-ALL progression in vivo

**Fig. 7:**
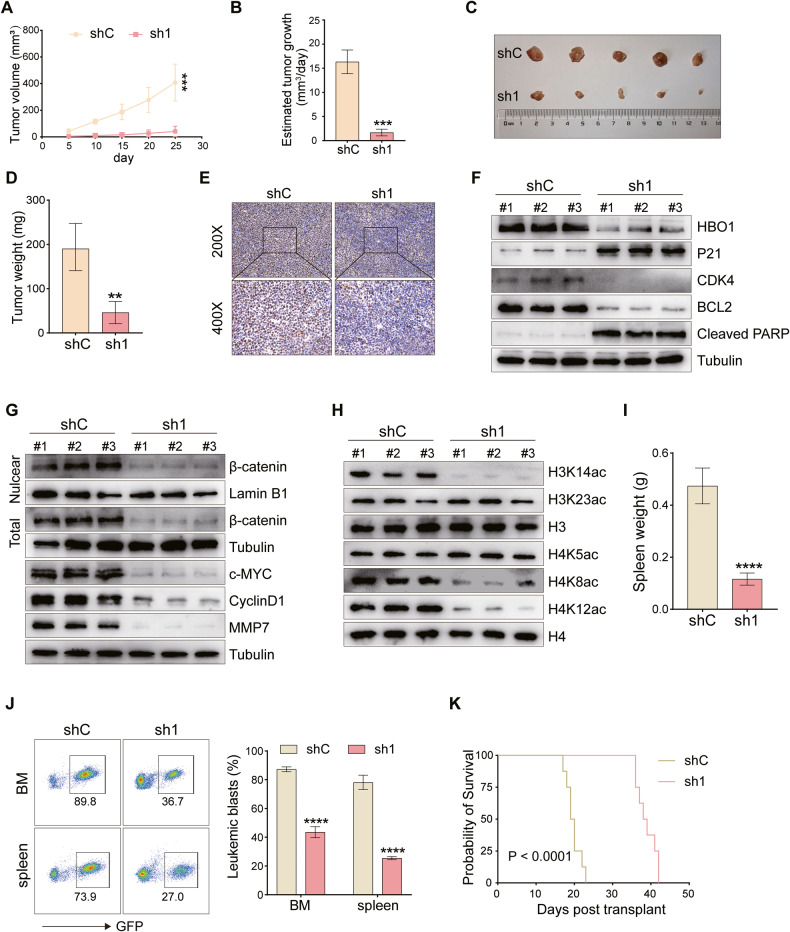
HBO1 knockdown suppresses B-ALL progression in vivo. **A**, **B** NALM-6 cells with or without HBO1 knockdown were implanted subcutaneously into B-NDG mice (*n* = 5 per group), and the tumor volume was recorded. **C**, **D** 25 days after subcutaneous inoculation of genetically modified NALM-6 cells, mice were sacrificed, tumor tissues were removed, then tumors were photographed and weighed. **E** Ki67 staining was performed to observe tumor proliferation after the removal of subcutaneous tumors in mice. **F**–**H** The subcutaneous tumors of mice in both groups injected with NALM-6/shC or NALM-6/shHBO1 cells were removed and ground, and then western blot assays were used to detect the expression of the listed proteins. **I**, **J** GFP-tagged NALM-6 cells with or without HBO1 knockdown were injected into B-NDG mice via the tail vein (*n* = 5 per group). 20 days later, the mice were sacrificed and the spleens and bone marrows were removed, then the spleens of the mice were weighed (**I**), and FACS analysis (**J**) was performed to detect tumor burden in the spleens and bone marrows. **K** Survival analysis of mice following tail vein injection of NALM-6 cells with or without HBO1 knockdown (*n* = 8 per group). Error bars indicate mean ± standard deviation (SD). Significance was tested by two-tailed unpaired student’s *t* tests (**A**, **B**, **D**, **I**, **J**) and log-rank test (**K**). **P < 0.01, ***P < 0.001, ****P < 0.0001.

We examined the potential effect of HBO1 on B-ALL cell growth in vivo. NALM-6 cells with HBO1 shRNA (sh1) or control vector (shC), were inoculated via *s.c*. injection to B-NGD mice. As demonstrated, HBO1-sh1 NALM-6 xenografts grew significantly slower than the control xenografts (Fig. 7A). When calculating the estimated daily tumor growth via the formula: (Tumor volume on Day 25)/25, we found that NALM-6 xenograft growth in vivo was inhibited by HBO1 knockdown (Fig. 7B). On Day 25, all tumors were separated and individually weighed. Tumors in HBO1-sh1 group mice were significantly lighter than those in the HBO1-shC group mice (Fig. 7C, D). We also investigated the cell proliferation rate by Ki67 staining of tumor samples. The HBO1-sh1 group exhibited a markedly reduced number of Ki67-positive cells compared with that of the HBO1-shC group (Fig. 7E). Western blot analysis of xenograft tumor issues showed that HBO1, cyclin-related protein CDK4, and anti-apoptotic protein BCL2 were decreased, but P21 and cleaved PARP protein expression were increased in HBO1-sh1 NALM-6 xenograft tissues (Fig. 7F), indicating the arrest of the cell cycle and the activation of apoptosis. The expression of β-catenin, a key molecule of the Wnt/β-catenin signaling pathway, and its downstream molecules c-MYC, Cyclin D1, and MMP7 was significantly decreased in the HBO1-sh1 group (Fig. 7G). In terms of histone acetylation levels, as shown in Fig. 7H, the acetylation of H3K14, H4K8, and H4K12 was significantly decreased in the NALM-6 xenografts with HBO1 shRNA. To better show the development of B-ALL in vivo, we injected NALM-6-shC or NALM-6-sh1 cells via the tail vein. Knockdown of HBO1 resulted in a reduction in spleen weight (Fig. 7I). As expected, HBO1 knockdown significantly inhibited the propagation of B-ALL cells and substantially prolonged survival in xenografted mice (Fig. 7J, K). Collectively, these data demonstrated that HBO1 knockdown suppressed B-ALL progression in vivo.

### WM-3835 exhibits significant anti-B-ALL activity

**Fig. 8:**
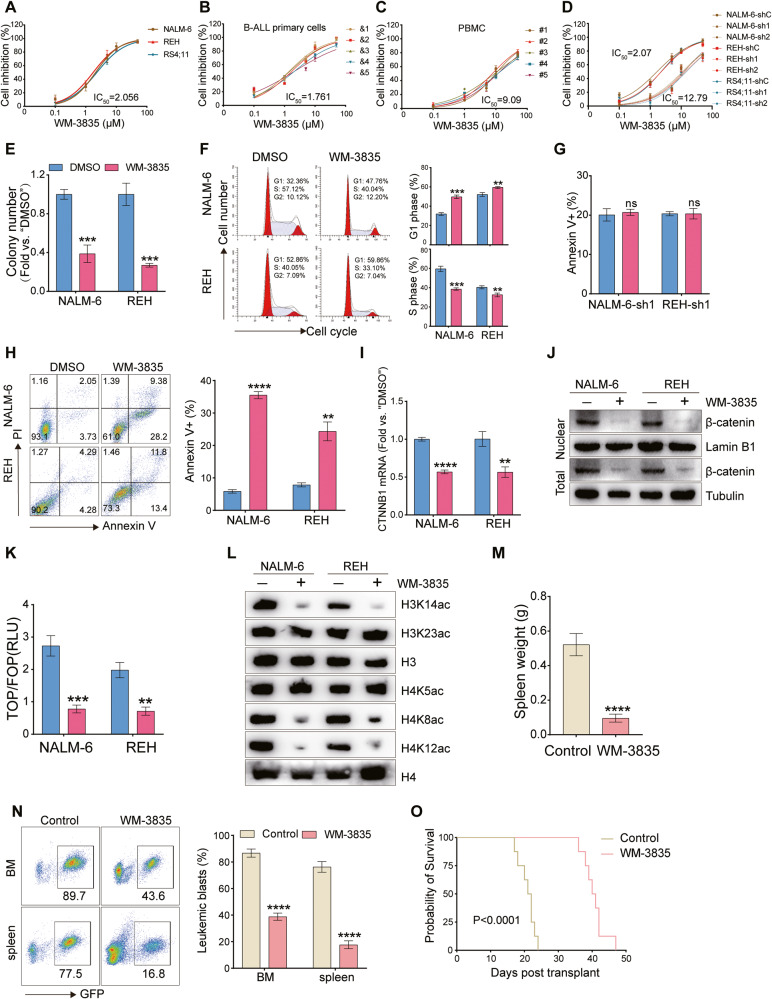
WM-3835 exhibits significant anti-B-ALL activity. **A**–**D** Dose-effect curves of the inhibitory action of WM-3835 on listed cells in vitro for 96 h. **E** The number of colonies of NALM-6 and REH cells treated with 0.1% DMSO or 2 μM WM-3835 and cultured for 10 days. **F** Cell cycle progression was detected by FACS (left) and statistically analyzed (right) after NALM-6 and REH cells were treated with 0.1% DMSO or 2 μM WM-3835 for 96 hours. **G** FACS analysis of the apoptotic HBO1 knockdown NALM-6 and REH cells treated with 0.1% DMSO or 2 μM WM-3835 for 96 h. **H**–**L** NALM-6 and REH cells were treated as **F**, then cell apoptosis (**H**), CTNNB1 mRNA (**I**), listed proteins (**J**, **L**), and the activity of Wnt signaling (**K**) were detected. **M**, **N** B-NDG mice (*n* = 5 per group) xenografted with GFP-tagged wild-type NALM-6 cells by tail vein injection were treated with WM-3835 (at 10 mg/kg body weight, daily for 2 weeks) or vehicle control through intraperitoneally injected. 20 days post xenograft, mice were sacrificed and spleens were removed and weighed (**M**), while tumor burden in the bone marrows and spleens (**N**) was detected by FACS. (**O**) Survival analysis of mice following tail vein injection of wild-type NALM-6 cells (*n* = 8 per group) and treated as **M**. Error bars indicate mean ± standard deviation (SD). Significance was tested by two-tailed unpaired student’s *t* tests (**E**–**I**, **K**, **M**, **N**) and log-rank test (**O**). ns no significance, **P < 0.01, ***P < 0.001, ****P < 0.0001. *n* = 3 per group (**A**–**I**, **K**).

Finally, we investigated the anti-B-ALL effect of WM-3835, a small molecule inhibitor of HBO1 [38]. We evaluated the impact of WM-3835 on the viability of B-ALL cell lines as well as B-ALL patient-derived primary cells and found that WM-3835 significantly inhibited their viability in a concentration-dependent manner (Fig. 8A, B). Interestingly, B-ALL patient-derived primary cells with high HBO1 expression were more sensitive to WM-3835 compared to those with relatively low HBO1 expression (Fig. S4). In contrast, WM-3835 did not affect the viability of normal human PBMC as well as HBO1 knockdown B-ALL cells (Fig. 8C, D), which suggested that WM-3835 was safe and acted against B-ALL by targeting HBO1. WM-3835 also reduced colony formation, and induced cell cycle G1-S arrest and apoptosis in NALM-6 and REH cells (Fig. 8E, F, H), without affecting the apoptosis of B-ALL cells with HBO1 knockdown (Fig. 8G). qRT-PCR and western blot experiments implied that WM-3835 decreased β-catenin expression at mRNA and protein levels in NALM-6 and REH cells (Fig. 8I, J). As shown in Fig. 8K, the activity of Wnt/β-catenin signaling was markedly reduced in NALM-6 and REH cells treated with WM-3835. We also investigated the effect of WM-3835 on histone acetylation of B-ALL cells. As expected, WM-3835 remarkably reduced acetylation levels of H3K14, H4K8, and H4K12 in NALM-6 and REH cells, except H3K23 and H4K5 (Fig. 8L). Finally, we investigated the in vivo anti-B-ALL effect of WM-3835 and showed that WM-3835 was able to significantly reduce spleen weight and tumor burden, as well as prolong the survival time of B-ALL xenografted mice (Fig. 8M–O).

## Discussion

In this study, we identified HBO1 as a potential therapeutic target for B-ALL, which was significantly upregulated and associated with poor survival in B-ALL patients. A novel mechanism by which HBO1 promoted B-ALL progression had also been elucidated. HBO1 promoted H3K14, H4K8, and H4K12 acetylation, leading to increased CTNNB1 expression and Wnt/β-catenin signaling activation, resulting in enhanced B-ALL carcinogenesis (Fig. S5).

Recent studies have reported that HBO1 was overexpressed in human malignancies and was associated with tumorigenesis and cancer progression [27, 28]. However, some researchers have also manifested the conclusion that HBO1, contrary to the above, does not promote cell proliferation, which may be associated with different tumor types [30, 31]. Therefore, it is meaningful to explore the role and mechanisms of HBO1 in specific types of cancer with different genetic backgrounds.

As demonstrated, HBO1 knockdown inhibited B-ALL cell viability, proliferation, and G1-S cycle progression, but activated apoptosis. HBO1 knockout by CRISPR/Cas9 also induced significant anti-B-ALL cell activity. In contrast, ectopic overexpression of HBO1 enhanced viability and proliferation but inhibited apoptosis in B-ALL cells. Experiments in vivo further manifested the inhibitory effect of HBO1 knockdown on tumor growth. Given the positive role of HBO1 in B-ALL progression, in-depth elucidation of the tumor-promoting mechanisms of HBO1 is a research topic worth exploring.

β-catenin is a pivotal regulatory molecule of the Wnt/β-catenin signaling pathway and is encoded by the CTNNB1 gene. Increasing evidence suggested that aberrant activation of Wnt/β-catenin signaling promoted the progression of multiple malignancies, including colorectal cancer [45], breast cancer [46], liver cancer [47], lung cancer [48], prostate cancer [49], AML [50], and B-ALL [51]. Aberrant activation of the Wnt/β-catenin signaling plays a vital role in the initiation and progression of cancers by promoting stemness, proliferation, cell motility, etc. [52]. Chen et al. and Wu et al. reported that HBO1 elevated the activity of Wnt/β-catenin signaling in bladder cancer and glioblastoma, but did not illuminate the specific mechanism by which HBO1 activated this pathway [29, 41].

In the present study, RNA sequencing results revealed that gene expression of the Wnt/β-catenin signaling was markedly altered following the knockdown of HBO1 in B-ALL, including the downregulation of CTNNB1. qRT-PCR, western blot, and immunofluorescence assays were performed to further validate the downregulation of β-catenin. Results of the TOP/FOP flash luciferase reporter assays demonstrated that the activity of Wnt/β-catenin signaling was attenuated in B-ALL after HBO1 knockdown. Following HBO1 knockdown in B-ALL, the expression of three downstream molecules of β-catenin was also downregulated, including c-MYC, Cyclin D1, and MMP7. Importantly, a positive correlation between HBO1 and CTNNB1 expression was observed in clinical B-ALL specimens. Next, the recombinant β-catenin plasmid was transiently transfected into the HBO1 knockdown B-ALL cells and the results manifested that the inhibitory effect of HBO1 knockdown on viability and proliferation, as well as apoptotic activation, was rescued. Moreover, the protein expression of c-MYC, Cyclin D1, and MMP7 after HBO1 knockdown was also elevated. These results implied that β-catenin might be the crucial molecule by which HBO1 enhanced the activity of the Wnt/β-catenin signaling pathway.

HBO1-containing complexes have been verified to acetylate histone H3 and H4, and the scaffold subunits BRPF and JADE determined the specificity of target histones. The BRPF-containing complex is responsible for acetylating H3K14 and K23, whereas the JADE-containing complex is responsible for acetylating H4K5, K8, and K12 [23]. HBO1 can acetylate histone and function as a regulator of gene expression [22]. In ovarian cancer, HBO1 preferentially acetylates histone H4 and is required for the expression of YAP1, an ovarian cancer oncogene and mechano-transductor signaling factor [53]. Au et al. found that acetyltransferase activation of HBO1 was essential for the proliferation of AML cells, as acetylated histone provided a platform for the recruitment of BRD4 and AF4 to gene promoters, including MEIS1, PBX3, and SENP6 [54]. MacPherson et al. identified that the HAT domain of HBO1 was essential for the acetylation of H3K14, and favored the processivity of RNA polymerase II to maintain high expression of key genes, including HOXA9 and HOXA10 that help maintain the functional properties of leukemia stem cells [38]. Our results revealed that HBO1 was responsible for the acetylation of H3K14, H4K8, and H4K12 in B-ALL, a pattern that has not been described before. Notably, the re-expression of HBO1-WT upregulated CTNNB1 transcription in HBO1 knockout cells and activated the Wnt/β-catenin signaling pathway, whereas the re-expression of HBO1 acetyltransferase activity mutant did not. CHIP-qPCR assays showed that the enrichment of HBO1 mediated H3K14, H4K8, and H4K12 acetylation on the promoter regions of the CTNNB1 gene. Our data demonstrated for the first time that the acetyltransferase activity of HBO1 is crucial for the activation of the Wnt/β-catenin signaling pathway. Finally, we investigated the antileukemic effect of WM-3835, a small molecule inhibitor of HBO1 [38]. We found that WM-3835 could inhibit B-ALL cell viability and colony formation, induce G1-S cycle arrest, and activate apoptosis. Remarkably, the activity of WM-3835 was ineffective in HBO1 knockdown B-ALL cells and was not cytotoxic to normal human PBMC, suggesting the targetability and safety of WM-3835. Furthermore, this HBO1 inhibitor significantly inhibited the activity of the Wnt/β-catenin signaling and the acetylation of H3K14, H4K8, and H4K12. At the same time, WM-3835 displayed significant anti-B-ALL efficacy in vivo. Therefore, targeting HBO1 by WM-3835 may be a novel strategy for the treatment of B-ALL.

In conclusion, we identified HBO1 as a potential target for the treatment of B-ALL. Our data revealed a novel mechanism that HBO1 acetylated H3K14, H4K8, and H4K12, and then activated the Wnt/β-catenin signaling, resulting in the progression of B-ALL. The role of HBO1 in B-ALL elucidated in this paper also contributes to a better understanding of the function of HBO1 in human disease.

## Supplementary information

Supplement materials

Reproducibility checklist

Uncropped western blots

## Data Availability

The datasets generated during and/or analyzed during the current study are available from the corresponding author upon reasonable request.
