# Supplementary material for: Histone acetylation by HBO1 (KAT7) activates Wnt/β-catenin signaling to promote leukemogenesis in B-cell acute lymphoblastic leukemia
Source: Cell Death Dis. 2023 Aug 4;14(8):498. doi: 10.1038/s41419-023-06019-0 (PMC10403501; doi:10.1038/s41419-023-06019-0)
Supplement: Supplementary file 1 — Supplement materials [file 41419_2023_6019_MOESM1_ESM.docx]

**Table S1. primer sequences utilized in this study.**

| **qRT-PCR** | Forward 5’-3’ | Reverse 5’-3’ |
| --- | --- | --- |
| β-actin | TGGCACCCAGCACAATGAA | CTAAGTCATAGTCCGCCTAGAAGCA |
| HBO1 | TCCATCTCAGGATGCCCACTGT | GTCATCTTGCCTGTGAGACAGC |
| CTNNB1 | CACAAGCAGAGTGCTGAAGGTG | GATTCCTGAGAGTCCAAAGACAG |
| **CHIP-qPCR** |  |  |
| CTNNB1 | AAATTCAAGCTGAACAGCCTGC | GCCGACCTTGTGGTCTGT |

**Table S2. Clinical information of the B-ALL samples used.**

| No. | Sex | Age | Time point | Blasts (%) | Gene mutation | HBO1 level (2^-ΔΔCT) | DFS(days) |
| --- | --- | --- | --- | --- | --- | --- | --- |
| 1 | Male | 27 | relapse | 83.5% | ETV-RUNX1 | 5.27 | 27 |
| 2 | Female | 24 | relapse | 33.0% | MLL-AF4 | 5.01 | 72 |
| 3 | Male | 20 | diagnosis | 94.5% | TEL-RUNEX1 | 4.22 | 87 |
| 4 | Female | 61 | relapse | 32.0% | BCR-ABL-P190,T315I | 4.08 | 99 |
| 5 | Male | 22 | diadnosis | 96.0% | TEL-ANL1,WT1 | 4.03 | 106 |
| 6 | Female | 32 | relapse | 67.0% | WT1/ABL | 3.88 | 118 |
| 7 | Male | 30 | relapse | 18.5% | BCR-ABL-P190 | 3.60 | 123 |
| 8 | Male | 54 | diagnosis | 83.7% | BCR-ABL | 3.50 | 144 |
| 9 | Male | 31 | relapse | 90.0% | BCR-ABL-P190 | 3.01 | 161 |
| 10 | Male | 20 | relapse | 7.2% | PTPN11,F594C,FAT3 | 1.34 | 192 |
| 11 | Male | 40 | relapse | 10.5% | IKZF1,MLL-AF4,WT1 | 1.25 | 202 |
| 12 | Male | 29 | relapse | 86.0% | TP53,PHF6 | 1.24 | 254 |
| 13 | Male | 20 | diagosis | 52.5% | EBF1-PDGFRB,FAT1 | 1.17 | 269 |
| 14 | Male | 44 | relapse | 78.5% | NOTCH | 1.16 | 329 |
| 15 | Male | 19 | relapse | 9.0% | MLL-PTD/ABL | 1.14 | 360 |
| 16 | Male | 23 | relapse | 94.5% | MYC, BCR/ABL1 | 1.14 | 691 |
| 17 | Female | 69 | relapse | 65.0% | WT1/ABL | 1.11 | 800 |
| 18 | Male | 22 | remission | 1.5% | FAT3,KMT2D | 2.93 | 99 |
| 19 | Female | 55 | remission | 1.0% | BCR/ABL | 2.91 | 66 |
| 20 | Male | 24 | relapse | 90.7% | CRLF2,IKZF1 | 2.78 | 172 |
| 21 | Female | 31 | remission | 1.0% | IKZF1,HOX11 | 2.65 | 157 |
| 22 | Female | 41 | relapse | 93.5% | BCR-ABL-P190 | 2.26 | 307 |
| 23 | Female | 58 | diagnosis | 65.0% | BCR-ABL-P190,IKZF1 | 2.14 | 293 |
| 24 | Female | 46 | remission | 2.0% | BCR-ABL-P190,EVI1 | 2.12 | 46 |
| 25 | Male | 55 | diagnosis | 57.2% | P210,TP53,IKZF1 | 1.74 | 15 |
| 26 | Male | 21 | diagnosis | 42.0% | IKZF1 | 1.67 | 462 |
| 27 | Male | 18 | diagnosis | 30.0% | TP53,CDKN2A,FAT1 | 1.56 | 131 |
| 28 | Male | 22 | remission | 2.0% | WT1,NRAS | 1.55 | 245 |
| 29 | Male | 70 | diagnosis | 82.0% | WT1,TP53,NF1 | 1.53 | 10 |
| 30 | Female | 27 | diagnosis | 89.8% | E2A-PBX1 | 1.44 | 650 |
| 31 | Male | 18 | diagnosis | 49.3% | NF1,PIK3CA | 1.40 | 172 |
| 32 | Male | 38 | diagnosis | 71.5% | BCR-ABL-P190 | 1.39 | 316 |
| 33 | Male | 19 | diagnosis | 61.0% | BCR-ABL-P190,IKZF1 | 1.10 | 203 |
| 34 | Male | 24 | remission | 0.5% | IKZF1,NUP214 | 1.09 | 669 |
| 35 | Male | 58 | relapse | 85.5% | KRAS, TP53 | 1.09 | 207 |
| 36 | Male | 34 | diagnosis | 93.5% | BCR-ABL-P190,IKZF1 | 1.01 | 314 |
| 37 | Male | 64 | diagnosis | 30.0% | TP53,FAT1 | 1.00 | 200 |
| 38 | Male | 27 | remission | 3.0% | BCR-ABL-P190,CREBBP | 0.99 | 508 |
| 39 | Female | 26 | diagnosis | 96.0% | WT1 | 0.98 | 347 |
| 40 | Female | 20 | diagnosis | 75.9% | TCF3-PBX1 | 0.95 | 134 |
| 41 | Male | 21 | diagnosis | 80.7% | FLT3,ZBTB7A,DPF2 | 0.86 | 825 |
| 42 | Female | 20 | diagnosis | 91.5% | BCR-ABL-P190 | 0.85 | 249 |
| 43 | Male | 35 | remission | 1.0% | SIL-TAL1,HOX11 | 0.83 | 748 |
| 44 | Female | 72 | diagnosis | 94.7% | CRLF2 | 0.82 | 117 |
| 45 | Male | 21 | relapse | 70.0% | IKZF1 | 0.82 | 221 |
| 46 | Male | 18 | diagnosis | 87.0% | IKZF3 | 0.82 | 146 |
| 47 | Male | 19 | diagnosis | 99.5% | KRAS,NF1,WT1 | 0.64 | 166 |

Abbreviation: DFS, Disease-free survival.


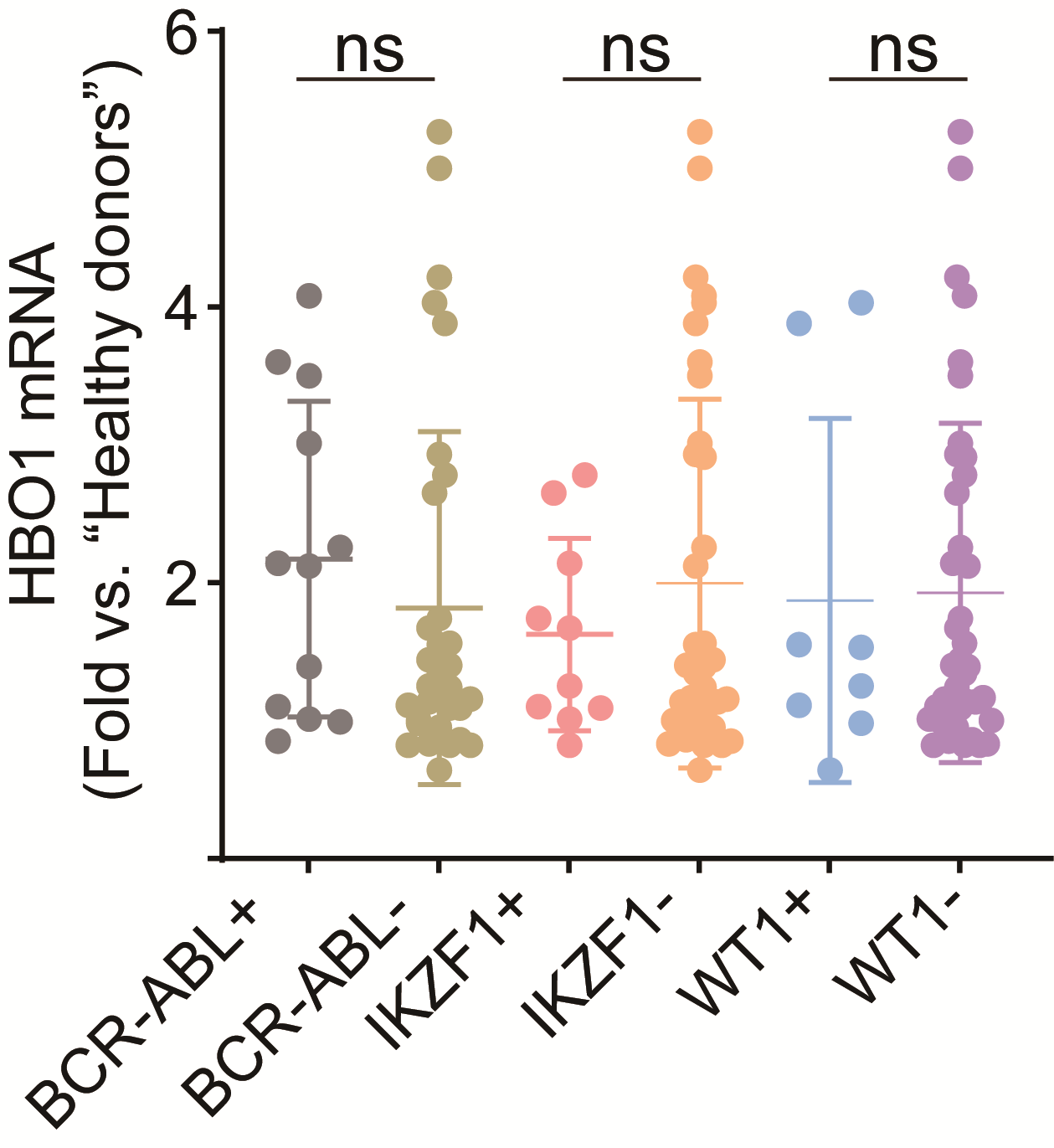


**Figure S1. HBO1 mRNA expression in B-ALL patients with different gene mutations.**

The expression of HBO1 mRNA in B-ALL patients with or without the listed gene mutation in our clinical sample data. Significance was tested by 2-tailed unpaired student’s t-tests. ns, no significance.


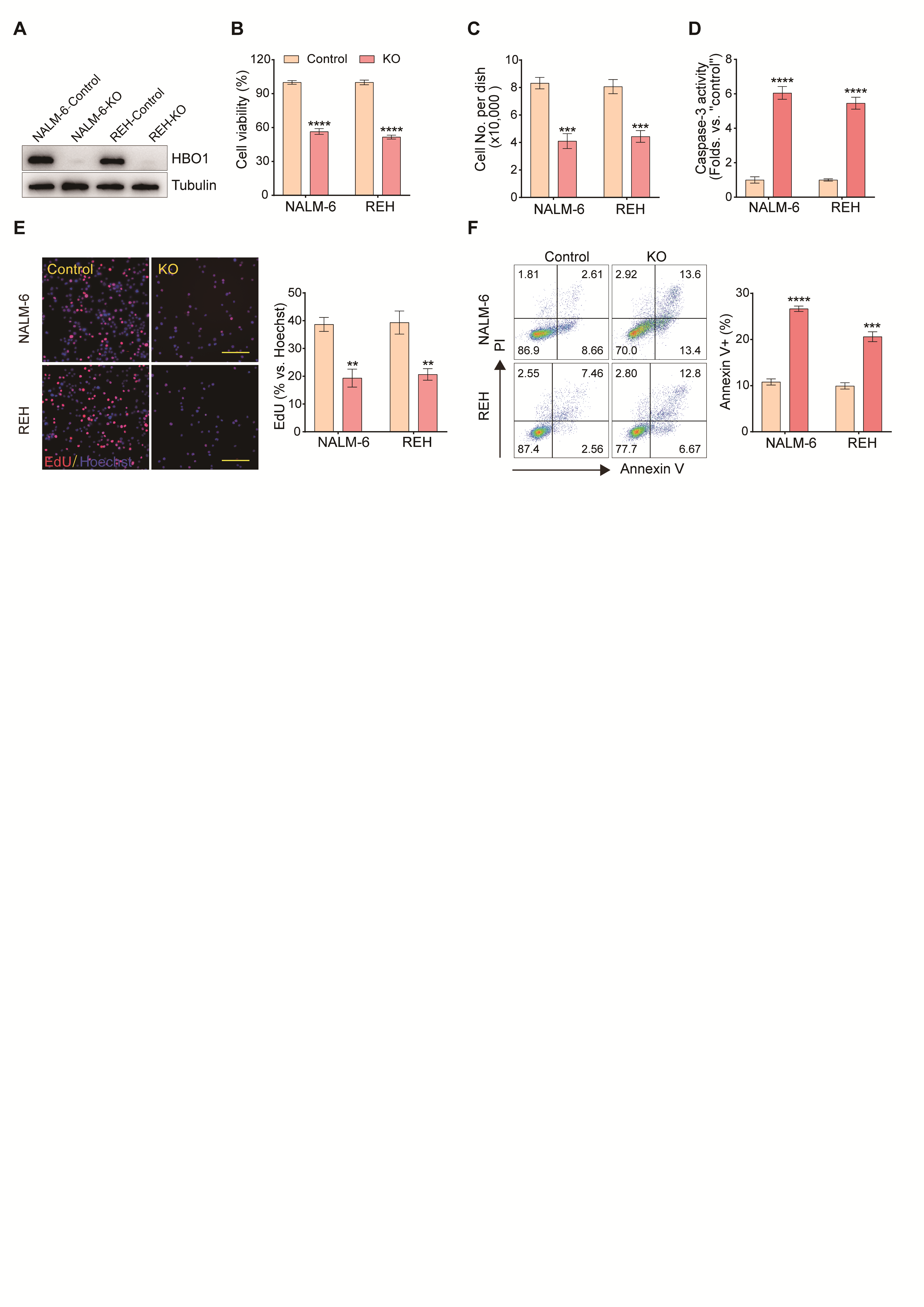


**Figure S2. HBO1 knockout by CRISPR/Cas9 induces significant anti-B-ALL cell activity.**

(**A**). Western blot assays were used to detect the expression of HBO1 protein in NALM-6 and REH cells with or without HBO1 knockout. (**B-C**). NALM-6 and REH cells with or without HBO1 knockout were cultured for 96 h and then cell viability (B) and number (C) was measured. (**D**). Statistical plots of caspase-3 activity in NALM-6 and REH cells cultured for 48 h with or without HBO1 knockout. (**E**). Nuclear EdU incorporation (left) and statistics (right) of NALM-6 and REH cells treated as (D). (**F**). FACS analysis (left) and statistics (right) of the apoptosis of NALM-6 and REH cells treated as (D). Scale bar = 100 μm (E). Error bars indicate mean ± standard deviation (SD). Significance was tested by 2-tailed unpaired student’s t-tests (B-F), ** *P* < 0.01, *** *P* < 0.001, **** *P* < 0.0001. n=3 per group (B-F).


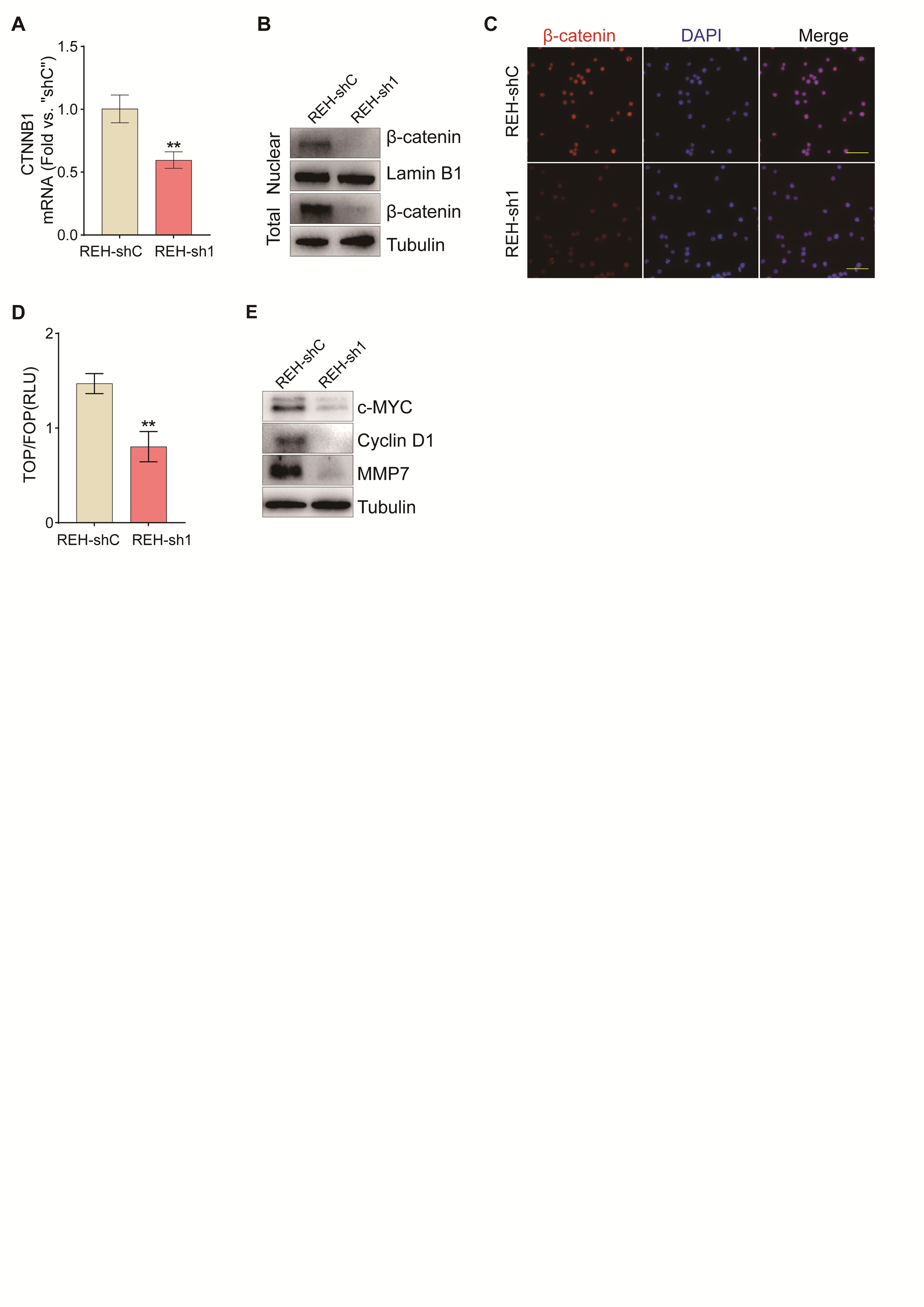


**Figure S3. Knockdown HBO1 inhibits the Wnt signaling pathway.**

(**A**-**B**). The expression of CTNNB1 mRNA (A) and β-catenin protein (B) in REH cells with or without HBO1 knockdown. (**C**). Immunofluorescence experiments were used to detect the expression of β-catenin protein in REH cells treated as (A). (**D**). The activity of Wnt signaling in REH cells treated as (A). (**E**). Listed proteins were detected in REH cells treated as (A) through western blot assays. Scale bar=100 μm (C). Error bars indicate mean ± standard deviation (SD). Significance was tested by 2-tailed unpaired student’s t-tests (A, D). ** *P* < 0.01. n=3 per group (A, D).


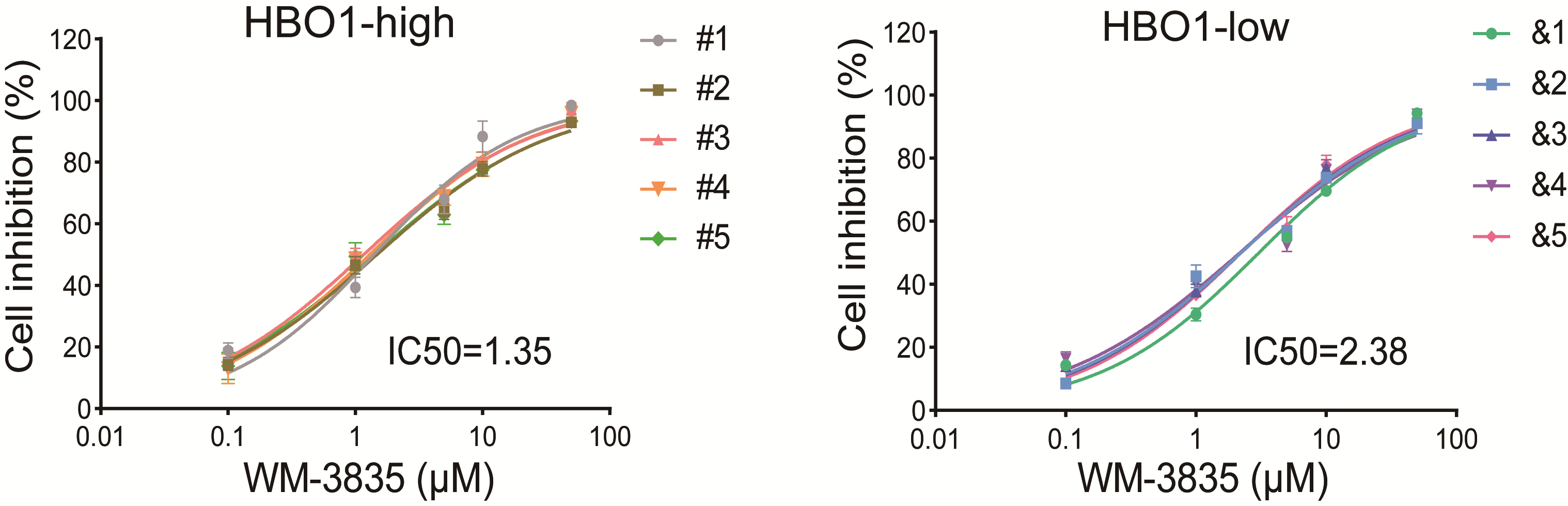


**Figure S4**. **B-ALL primary cells with high HBO1 expression are more sensitive to WM-3835.**

Dose-effect curves of the inhibitory action of WM-3835 on B-ALL primary cells from B-ALL patients with HBO1 high (left) or low (right) expression *in vitro* for 96 h.


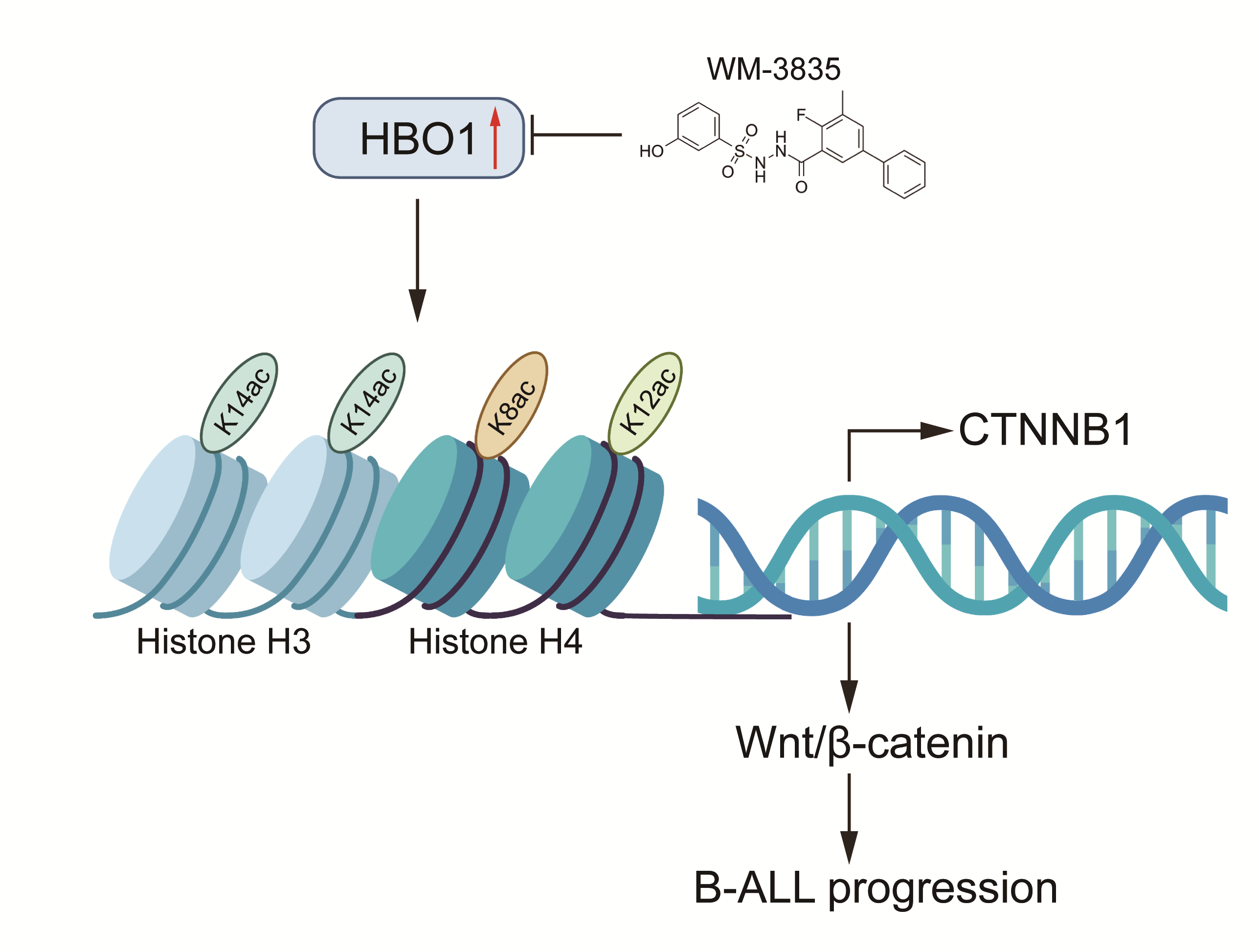


**Figure S5. A working model for HBO1-regulated B-ALL progression.**

HBO1 was upregulated in B-ALL cells and then boosted the acetylation of H3K14, H4K8, and H4K12, thereby facilitating the transcription of CTNNB1, ultimately leading to activation of the Wnt/β-catenin signaling pathway as well as the progression of B-ALL. However, HBO1-targeted inhibitor WM-3835 could inhibit this effect.
