# Supplementary material for: Histone acetylation by HBO1 (KAT7) activates Wnt/β-catenin signaling to promote leukemogenesis in B-cell acute lymphoblastic leukemia
Source: Cell Death Dis. 2023 Aug 4;14(8):498. doi: 10.1038/s41419-023-06019-0 (PMC10403501; doi:10.1038/s41419-023-06019-0)

Fig. 1C

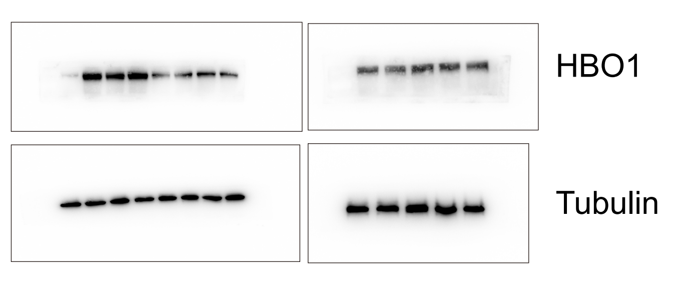

Fig. 1E

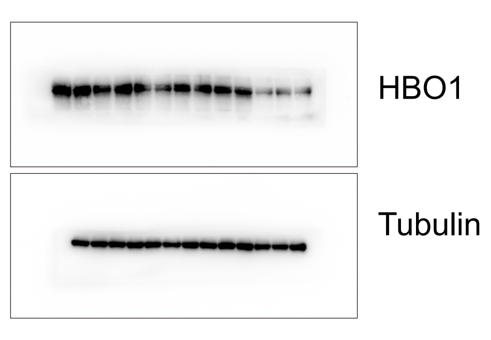

Fig. 2B

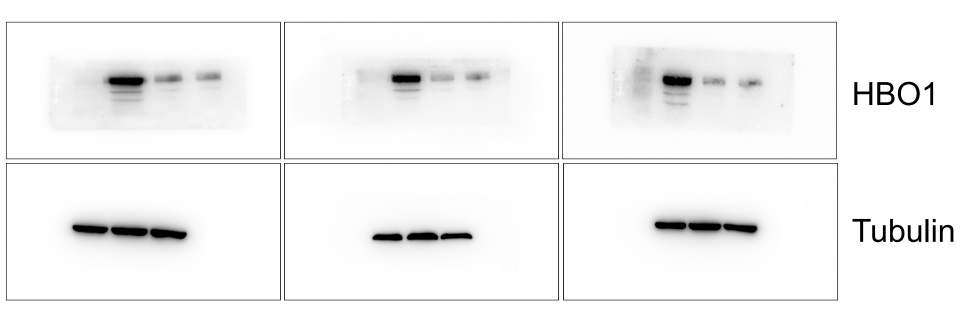

Fig. 2H

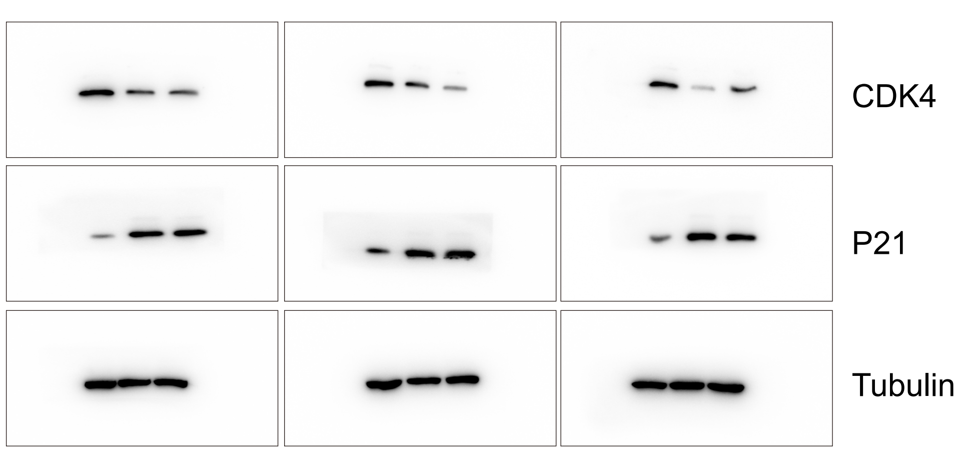

Fig. 3C

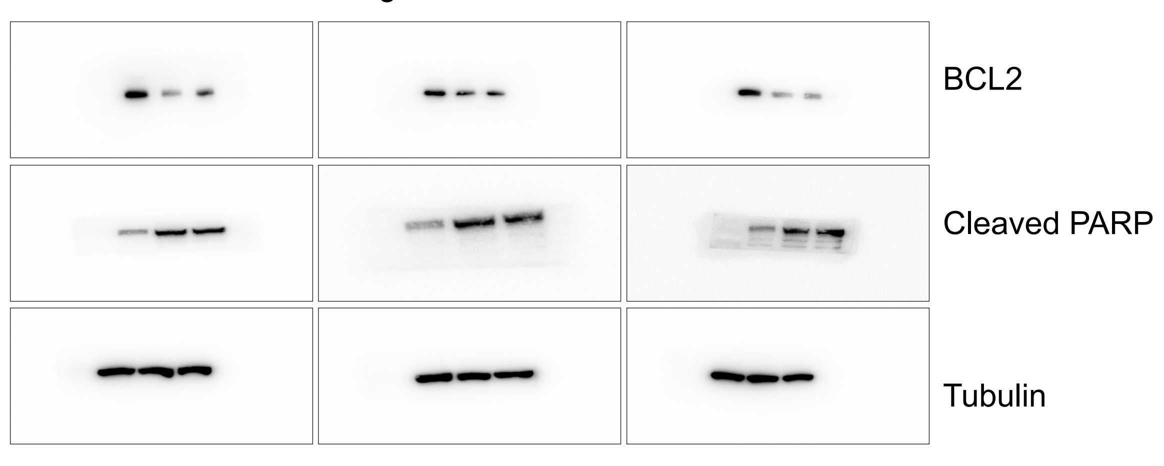

Fig. 4B

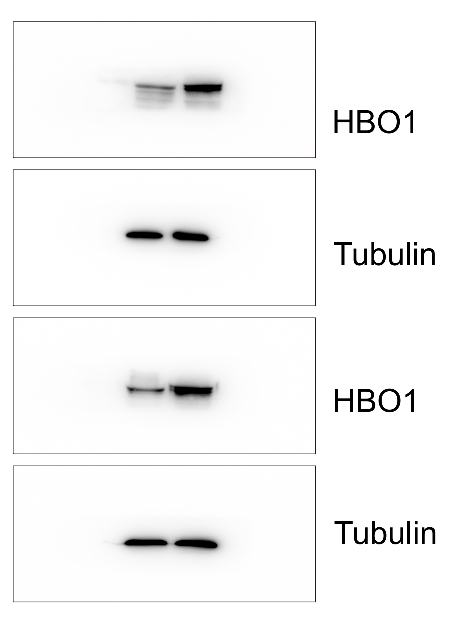

Fig. 4G

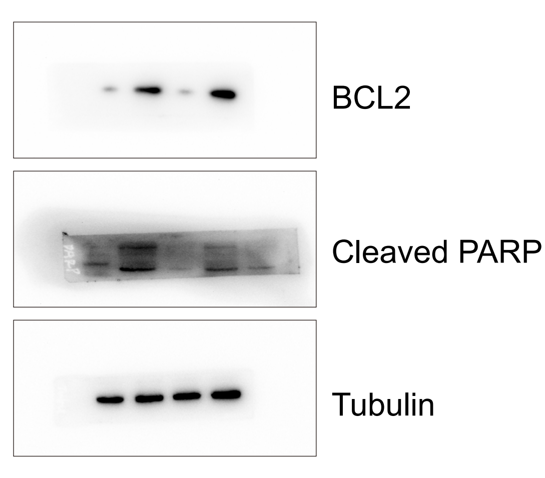

Fig. 5E

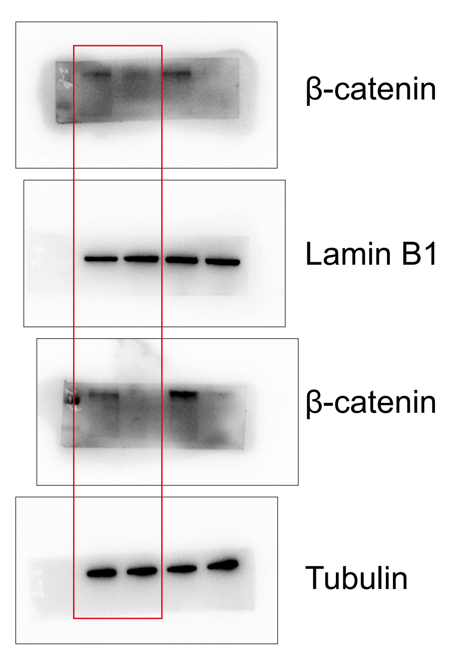

Fig. 5H

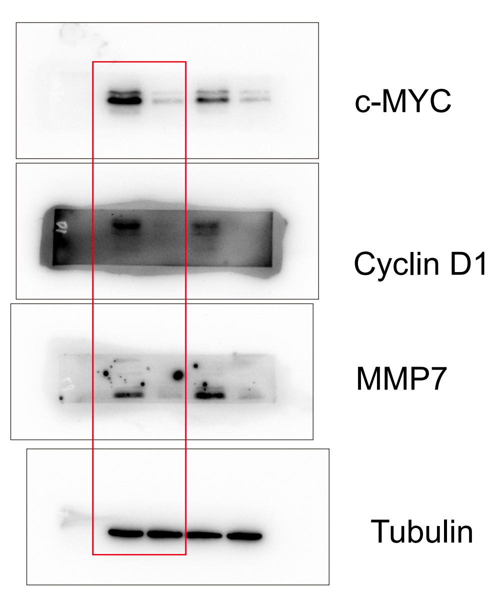

Fig. 5K

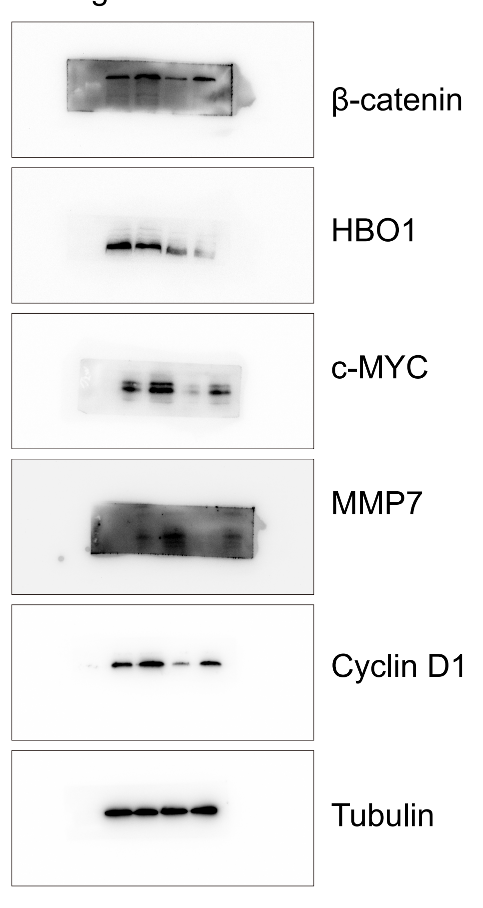

Fig. 6A

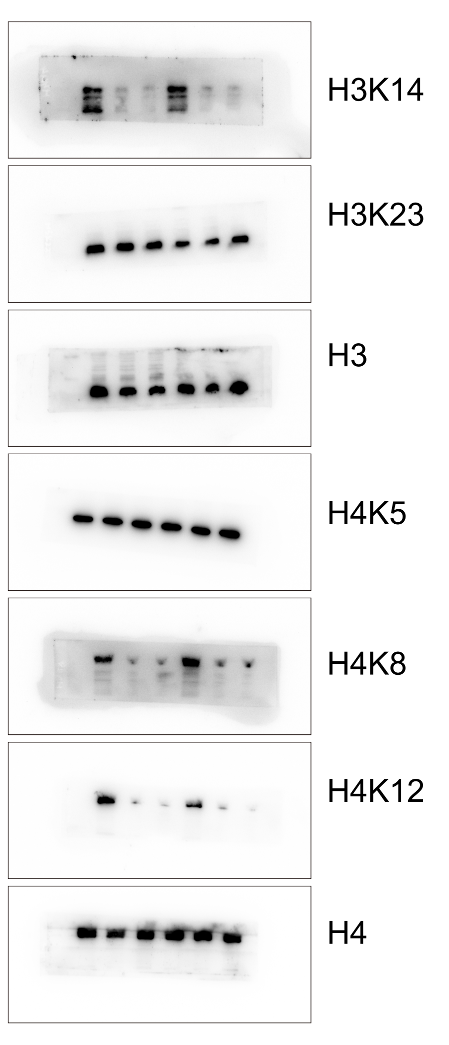

Fig. 6C

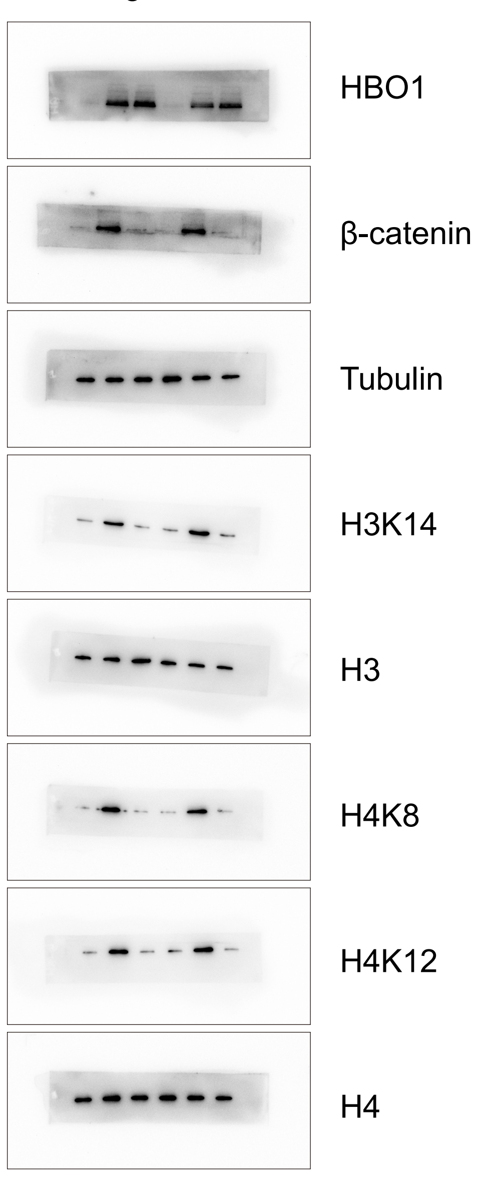

Fig. 7F

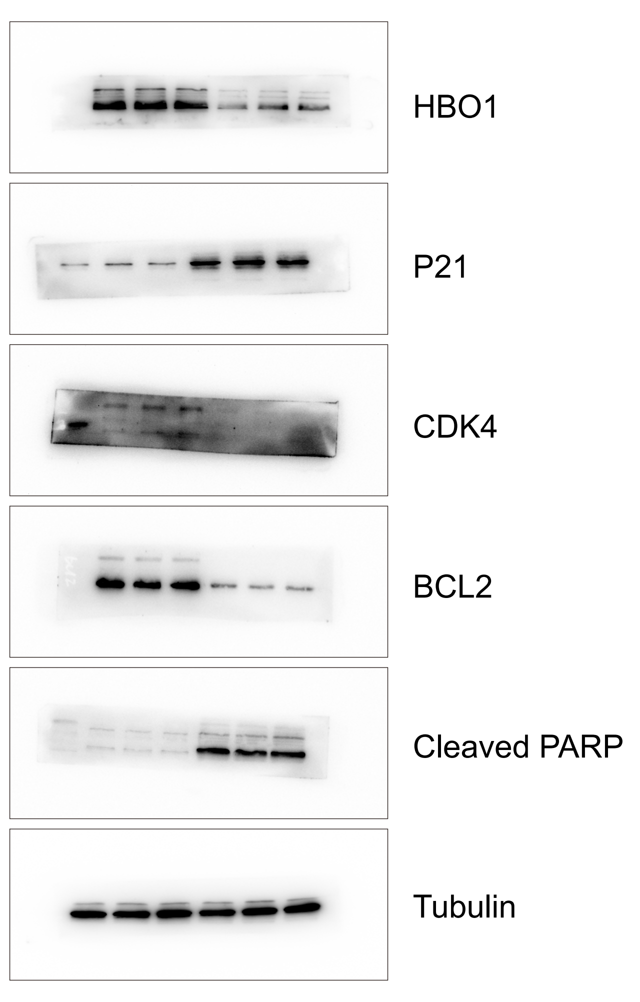

Fig. 7G

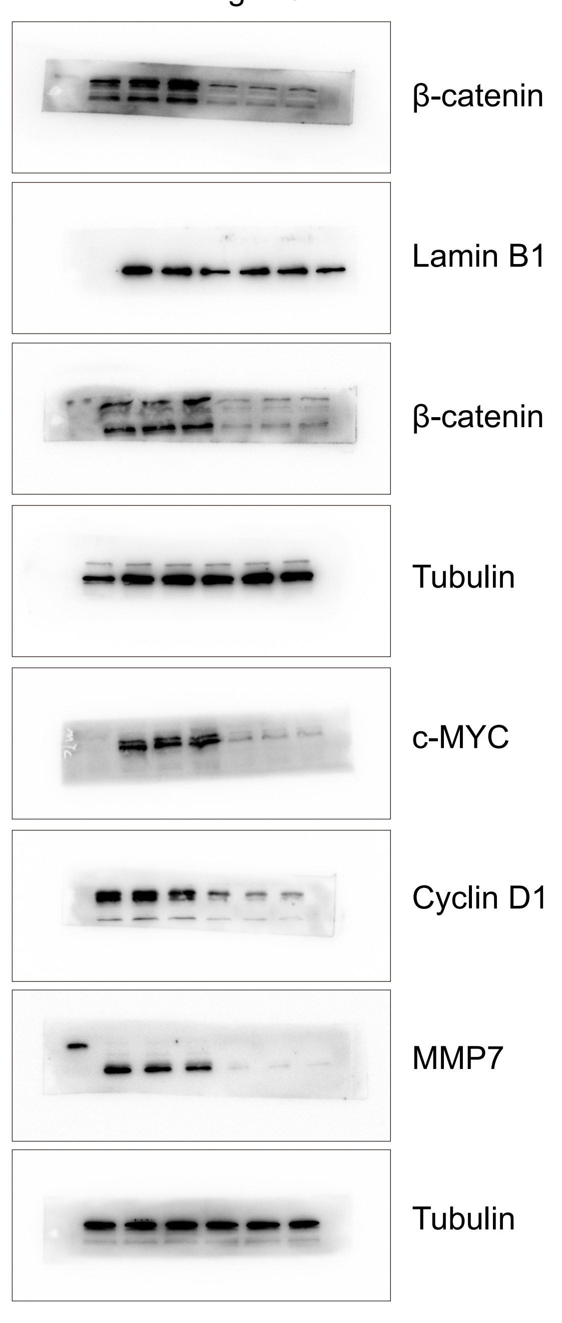

Fig. 7H

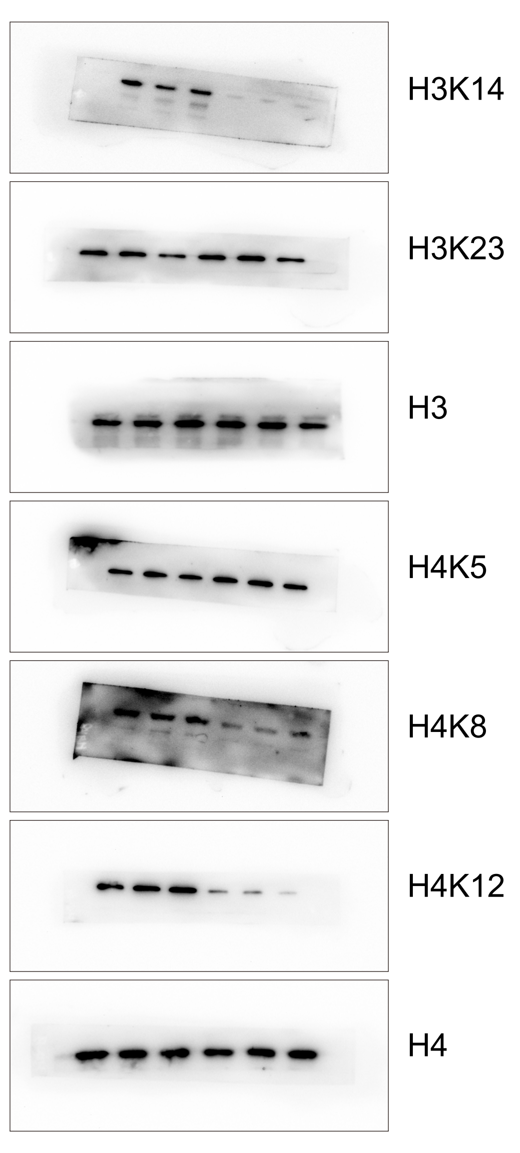

Fig. 8J

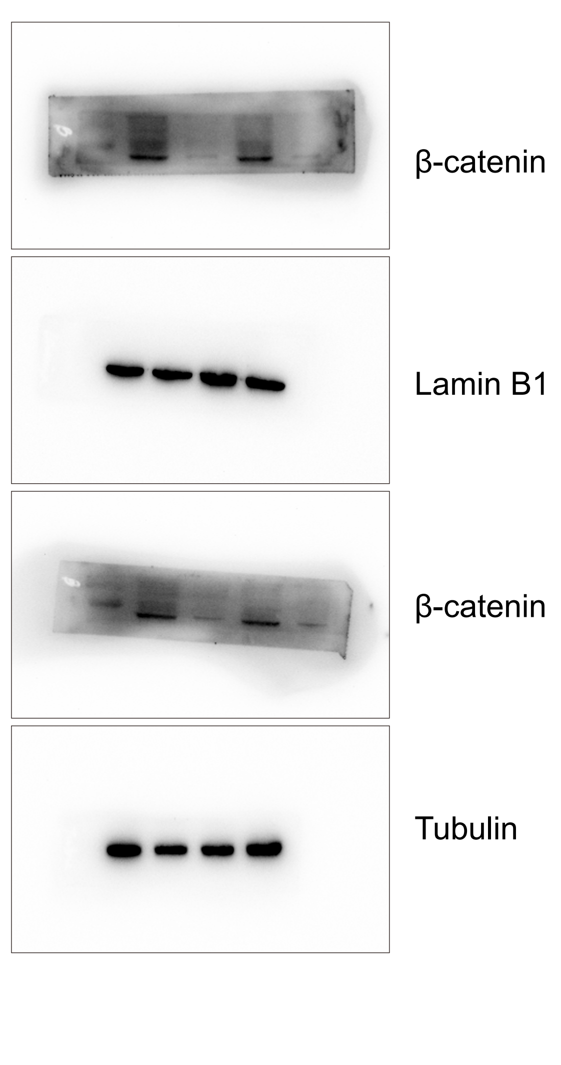

Fig. 8L

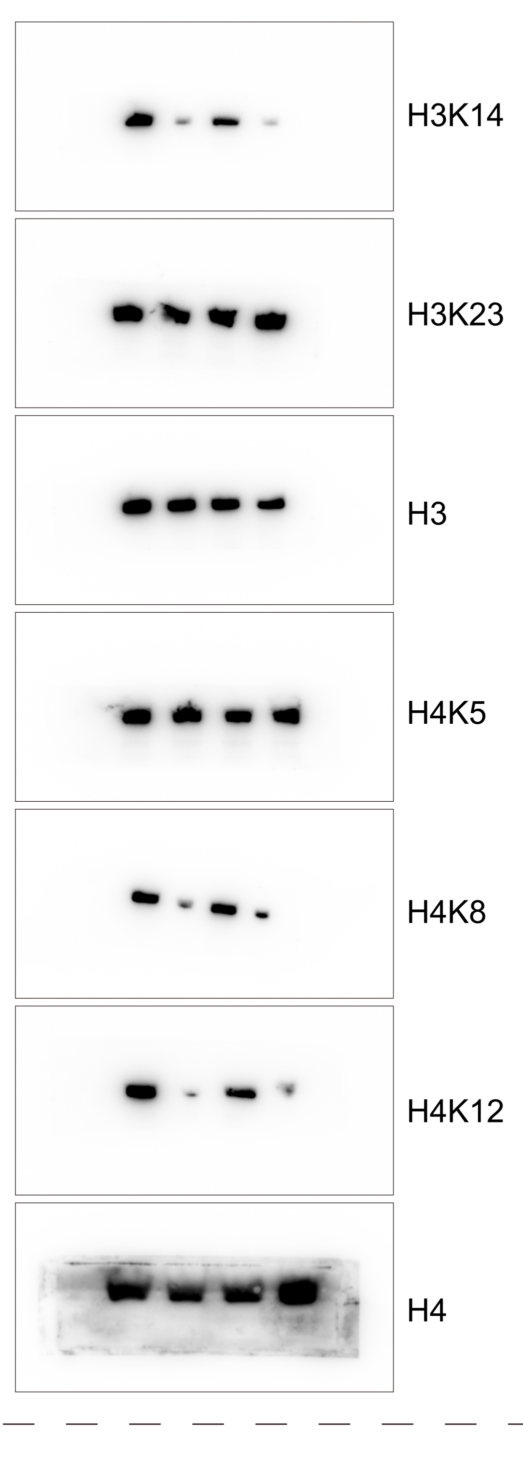

Fig. S2A

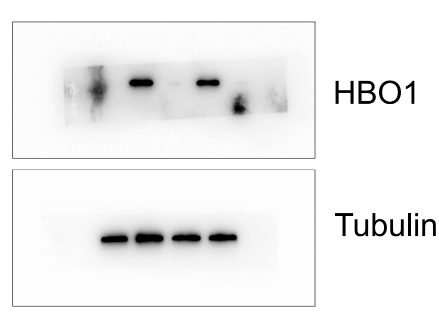

Fig. S3B

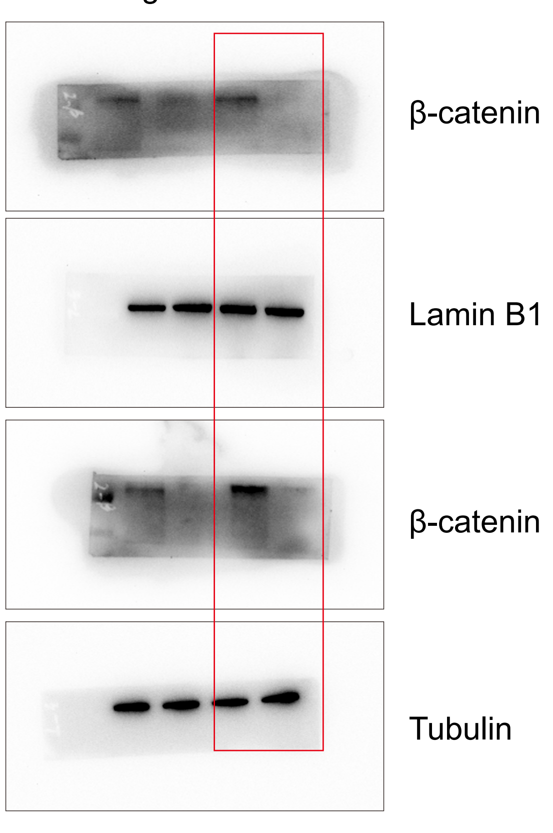

Fig. S3E

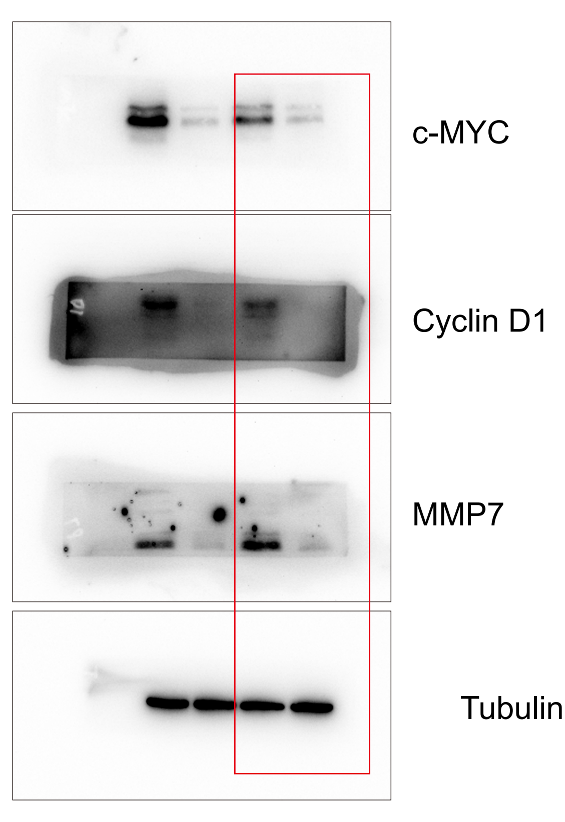

Supplement: Supplementary file 3 — Uncropped western blots [file 41419_2023_6019_MOESM3_ESM.pdf]
